# Photonics on a Budget: Low-Cost Polymer Sensors for a Smarter World

**DOI:** 10.3390/mi16070813

**Published:** 2025-07-15

**Authors:** Muhammad A. Butt

**Affiliations:** Institute of Microelectronics and Optoelectronics, Warsaw University of Technology, Koszykowa 75, 00-662 Warsaw, Poland; ali.butt@pw.edu.pl

**Keywords:** polymer photonic sensors, cost-effective, roll-to-roll, imprint fabrication

## Abstract

Polymer-based photonic sensors are emerging as cost-effective, scalable alternatives to conventional silicon and glass photonic platforms, offering unique advantages in flexibility, functionality, and manufacturability. This review provides a comprehensive assessment of recent advances in polymer photonic sensing technologies, focusing on material systems, fabrication techniques, device architectures, and application domains. Key polymer materials, including PMMA, SU-8, polyimides, COC, and PDMS, are evaluated for their optical properties, processability, and suitability for integration into sensing platforms. High-throughput fabrication methods such as nanoimprint lithography, soft lithography, roll-to-roll processing, and additive manufacturing are examined for their role in enabling large-area, low-cost device production. Various photonic structures, including planar waveguides, Bragg gratings, photonic crystal slabs, microresonators, and interferometric configurations, are discussed concerning their sensing mechanisms and performance metrics. Practical applications are highlighted in environmental monitoring, biomedical diagnostics, and structural health monitoring. Challenges such as environmental stability, integration with electronic systems, and reproducibility in mass production are critically analyzed. This review also explores future opportunities in hybrid material systems, printable photonics, and wearable sensor arrays. Collectively, these developments position polymer photonic sensors as promising platforms for widespread deployment in smart, connected sensing environments.

## 1. Introduction

The global demand for real-time, distributed, and cost-effective sensing technologies has grown rapidly in recent years, driven by applications in environmental monitoring, healthcare diagnostics, food safety, structural health, and industrial process control [1,2]. The rise of the Internet of Things (IoT) [3,4] and Industry 4.0 [5] has further intensified the need for sensor platforms that can be produced at low cost, deployed over large areas, and integrated seamlessly into smart systems [6]. Optical sensors have attracted significant interest in this context because of their unique advantages, including high sensitivity, immunity to electromagnetic interference, potential for remote sensing, and compatibility with multiplexing techniques [7,8,9,10].

Despite these strengths, conventional integrated photonic sensor platforms typically based on silicon [11,12], III-V semiconductors [13], or glass [14] face inherent limitations when it comes to large-area and low-cost manufacturing. The high capital costs associated with semiconductor fabrication, the rigidity of inorganic materials, and the complexities of scaling up these technologies to large surfaces have restricted their applicability in scenarios where affordability, mechanical flexibility, and scalability are critical [15]. This has created a pressing need for alternative materials and fabrication approaches capable of meeting the growing demands of modern sensing applications [16,17,18,19].

Polymer-based integrated photonic sensors have emerged as a highly promising solution to these challenges [20,21,22]. Polymer materials offer a unique combination of low cost, mechanical flexibility, and ease of processing that makes them particularly well-suited for large-area and scalable sensing technologies [23]. In addition to being inexpensive and lightweight, polymers can be processed using high-throughput, large-area fabrication techniques such as roll-to-roll (R2R) manufacturing [24,25], nanoimprint lithography (NIL) [26], soft lithography [27], and inkjet or 3D printing [28]. Their flexibility allows them to conform to curved and irregular surfaces, enabling new form factors such as wearable and skin-mounted sensors [29]. Moreover, polymers can be chemically tailored or functionalized with embedded dyes, nanoparticles, or biomolecules to create active sensing elements within the photonic structures themselves [30].

The optical properties of polymers, including their refractive indices, transparency in relevant wavelength ranges, and capacity for birefringence or nonlinearity, can be tuned to suit a wide variety of photonic device designs [31]. Polymer-based photonic waveguides [32], Bragg gratings [33], photonic crystal slabs [34], resonators [35], and interferometers [36] have all been explored as components for integrated sensors, with applications in chemical detection, biosensing, environmental monitoring, and beyond [20].

At the same time, polymer photonic sensors face important technical challenges. These include issues related to environmental stability, such as sensitivity to humidity, temperature, and ultraviolet light, as well as questions of long-term durability and integration with electronics for signal readout and processing [37]. Nonetheless, recent advances in polymer chemistry, hybrid material systems, and innovative fabrication methods are helping to overcome these limitations and are bringing the field closer to delivering scalable, low-cost, large-area photonic sensing solutions [38].

This review presents a comprehensive and up-to-date analysis of polymer-based large-area integrated photonic sensors, focusing on their material foundations, scalable fabrication processes, structural architectures, and application domains. Section 2 details the optical, mechanical, and chemical properties of key polymers such as PMMA, SU-8, polyimides, COC, and PDMS, emphasizing their suitability for integrated sensing applications. Section 3 explores scalable fabrication strategies, including NIL, soft lithography, R2R processing, and additive manufacturing, highlighting their compatibility with high-throughput, large-area device production. Section 4 delves into critical photonic structures such as waveguides, Bragg gratings, photonic crystals, resonators, and interferometers, examining their operating principles, integration techniques, and sensing performance. Section 5 surveys practical applications across environmental monitoring, biomedical diagnostics, and structural health monitoring, showcasing the real-world impact of polymer photonic sensors. Section 6 addresses major challenges, including environmental stability, reproducibility, and photonic–electronic integration, while also identifying opportunities in hybrid material systems and co-packaged optics. Section 7 offers a forward-looking perspective on emerging trends such as smart packaging, wearable sensor arrays, printable photonics and polymer photonics for environmental resilience and harsh conditions. Finally, Section 8 synthesizes the insights gained, reaffirming the potential of polymer photonic technologies to drive the future of low-cost, high-performance, and scalable sensing systems in diverse and connected environments.

## 2. Polymer Materials for Large-Area Photonic Sensors

The selection of polymer materials plays a critical role in determining the performance, scalability, and application potential of large-area integrated photonic sensors [39]. Polymers offer a broad palette of optical, mechanical, and chemical properties that can be tailored through molecular design, blending, or incorporation of functional additives [40]. This versatility makes them particularly attractive for photonic sensing platforms aimed at large-area, low-cost, and flexible implementations [41]. The overview of polymer materials and their characteristics for photonic sensors is presented in Table 1.

Among the most widely used polymer materials in integrated photonic sensors is polymethyl methacrylate (PMMA) [42]. PMMA is favored for its excellent optical transparency in the visible and near-infrared regions, low cost, and ease of processing. Its refractive index typically ranges from 1.48 to 1.50 at visible wavelengths, making it compatible for use as a cladding or waveguiding material when paired with higher-index functional layers or substrates [43]. PMMA is also amenable to NIL and other patterning techniques that are suitable for large-area fabrication [21].

SU-8, an epoxy-based negative photoresist, is another common material used in polymer photonics [44]. SU-8 offers a higher refractive index, typically around 1.57, and forms high-aspect-ratio structures with excellent mechanical stability after crosslinking. This makes it suitable for creating precise, high-resolution photonic structures such as waveguides, resonators, and gratings [45]. SU-8 is compatible with standard photolithography and imprinting processes and has been widely used in both planar and three-dimensional polymer photonic devices [46,47].

Polyimides represent another important class of polymer materials for photonic sensing applications [48]. Known for their outstanding thermal stability, chemical resistance, and mechanical robustness, polyimides can operate in demanding environments such as high-temperature or chemically aggressive conditions. Their refractive index typically lies between 1.65 and 1.70, offering strong optical confinement when used in waveguide structures. In addition, polyimides can be spin-coated or printed over large areas and patterned using a variety of techniques [49].

Cyclic olefin copolymers (COCs) have attracted attention for their excellent optical clarity, low birefringence, and low moisture absorption [50]. Their refractive index, around 1.53, and their compatibility with R2R processing make them suitable for large-area photonic sensors, particularly where low optical loss and environmental stability are required. COCs are also biocompatible, making them attractive for medical and bio-integrated photonic devices [51].

Polydimethylsiloxane (PDMS) is a highly flexible, elastomeric polymer widely employed in soft photonics [52]. PDMS has a refractive index of 1.41 and is valued for its optical transparency in the visible and near-infrared ranges, its ability to form conformal contact with surfaces, and its biocompatibility [53]. It is extensively used in microfluidics-integrated photonic sensors and flexible optical components [54]. Furthermore, PDMS can serve as both a structural material and a substrate for embedding other photonic elements [55].

The optical properties of these polymer materials, particularly refractive index, transparency, and optical loss, are key to their function in photonic sensors. The refractive index determines the guiding and confinement of light within waveguides or resonant structures [56,57]. Transparency across relevant spectral ranges ensures minimal absorption loss, which is crucial for sensitive detection. Most of these polymers exhibit low absorption loss in the visible and near-infrared regions, although scattering loss due to surface roughness or inhomogeneities can be a concern, especially for structures fabricated over large areas [58].

Beyond their intrinsic optical properties, polymers provide exceptional opportunities for functionalization. Polymers can be doped or blended with a variety of active materials to impart specific sensing capabilities [59,60]. For instance, nanoparticles such as gold, silver, copper, or quantum dots can be embedded into polymer matrices to enable plasmonic or enhanced fluorescence-based sensing [61,62,63]. Similarly, polymers can be loaded with responsive dyes that change their optical properties in response to environmental stimuli such as pH, temperature, or specific analytes [64]. Surface functionalization of polymer photonic structures with biomolecules, including antibodies, enzymes, or nucleic acids, can transform passive optical devices into highly selective biosensors [65]. The ability to integrate functional components directly into or onto the polymer matrix opens up a wide design space for realizing sensitive, specific, and tunable photonic sensors suitable for large-area applications.

**Table 1:** Overview of polymer materials and their characteristics for photonic sensors.

Polymer Material	Typical Refractive Index (at 633–1550 nm)	Transparency Range	Key Features	Role in Photonic Sensors
PMMA (Polymethyl methacrylate) [42,66,67]	1.48–1.50	Visible to NIR	Low cost, good optical clarity, easy patterning (e.g., nanoimprint)	Cladding layers, waveguides, and disposable sensor substrates
SU-8 [44,47]	~1.57	Visible to NIR	High mechanical stability, high-aspect-ratio patterning, epoxy-based	Waveguides, resonators, grating structures, and microfluidic-integrated sensors
Polyimide [48,49]	1.65–1.70	Visible to NIR	Excellent thermal and chemical stability, robust mechanical properties	Waveguides for harsh environments, chemically resistant sensors
COC (Cyclic Olefin Copolymer) [20,49,51]	~1.53	Visible to NIR	Low moisture absorption, low birefringence, and biocompatibility	Large-area substrates, low-loss waveguides, bio-integrated sensors
PDMS (Polydimethylsiloxane) [52,53,54]	~1.41	Visible to NIR	High flexibility, biocompatibility, transparent elastomer	Flexible sensors, microfluidic-integrated photonics, tunable structures

While the optical, mechanical, and chemical properties of polymer materials have been extensively characterized, it is equally critical to examine how these attributes specifically support the development of large-area photonic sensors [68]. For instance, PMMA’s excellent optical transparency and compatibility with NIL enable the replication of fine photonic features over expansive substrates at low cost, making it suitable for disposable, large-area sensor arrays [69]. SU-8, known for its high mechanical stability and high-aspect-ratio patterning, offers structural robustness over wide surfaces, which is beneficial for maintaining device fidelity in scaled-up systems [70]. However, its rigidity may require careful handling when used on flexible substrates. Polyimides, with their superior thermal and chemical resistance, are ideal for harsh environments and can be uniformly spin-coated across large areas, supporting scalable fabrication while ensuring environmental durability [71]. COCs with their low moisture absorption, optical clarity, and R2R compatibility are well-positioned for roll-to-roll processing, a key enabler of high-throughput large-area production [72]. Meanwhile, PDMS, due to its flexibility and conformal contact with curved surfaces, facilitates soft lithography on non-planar substrates, making it indispensable for wearable or skin-mounted sensor platforms [73]. When considered in the context of large-area applications, these material characteristics collectively underscore the unique advantages of polymers in enabling scalable, low-cost, and mechanically adaptable photonic devices. As such, the strategic selection and engineering of polymer materials are essential not only for achieving optimal photonic performance but also for the successful implementation of high-performance sensors across expansive and non-uniform surfaces.

In addition to well-established polymers as discussed in this section, several emerging polymer systems are gaining attention for their potential in large-area photonic sensing applications. High-index polymers such as polybenzoxazoles (PBOs) [74] and perfluorinated polymers (e.g., CYTOP variants) [75] offer ultra-low optical loss and high thermal stability, making them suitable for next-generation photonic waveguides. Organic–inorganic hybrid materials based on sol–gel chemistry or ORMOCER^®^ frameworks provide a tunable balance of optical clarity, mechanical strength, and functionalization potential, which is especially relevant for multi-modal sensing platforms [76]. Additionally, biodegradable and biocompatible polymers like silk fibroin and cellulose derivatives emerge in wearable and transient sensor designs, particularly in biomedical or environmental monitoring scenarios [77]. Functionalized conjugated polymers, including polythiophenes and polyfluorenes, are also being explored for their intrinsic optical responsiveness and compatibility with printable electronics [78]. These emerging materials, while still under investigation, hold significant promise for expanding the design space of large-area polymer photonic sensors, especially in contexts requiring flexibility, sustainability, or advanced optical functionalities.

## 3. Fabrication Techniques Enabling Low Cost and Large Area

The ability to produce photonic and nanostructured devices over large areas at low cost is essential for advancing applications in displays, sensors, photovoltaics, and optical coatings. Conventional high-resolution methods such as electron beam lithography (EBL) offer unmatched precision but are prohibitively expensive and slow when scaled to large areas [79]. As a result, several alternative fabrication techniques have emerged that provide a favorable balance of cost, throughput, and resolution, making them suitable for commercial-scale production. Key among these are NIL, soft lithography, R2R processing, and emerging printing techniques like inkjet and 3D printing (Table 2).

Each of these fabrication methods offers distinct advantages depending on the target application. NIL enables nanoscale precision but requires mold fabrication, making it ideal for high-resolution photonic structures. Soft lithography provides a low-cost, adaptable approach for patterning on flexible or curved substrates. R2R processing is unmatched in scalability and throughput for large-area devices. Meanwhile, inkjet and 3D printing deliver design flexibility and rapid prototyping capabilities, though typically with lower resolution. This diversity of approaches allows for tailored manufacturing strategies based on specific performance, scalability, and cost considerations.

### 3.1. Nanoimprint Lithography (NIL)

NIL is a promising high-resolution technique that enables direct physical replication of nanoscale patterns [24]. In NIL, a hard mold typically made of quartz or silicon and pre-patterned with nanoscale features is pressed into a resist-coated substrate, imprinting its structure [80]. The resist is then cured (thermally or with UV light), and the mold is released, leaving behind the desired pattern [81]. NIL can achieve sub-10 nm resolution and is highly versatile, supporting both rigid and flexible substrates [82]. It is well-suited for producing nanophotonic devices, metasurfaces, and anti-reflective coatings [83,84]. However, the high cost and complexity of mold fabrication, potential issues with mold wear, and challenges in aligning multilayer structures are limitations that must be managed, particularly for complex or multilayer devices [85].

Park et al. demonstrated a straightforward, single-step method for fabricating flexible ridge waveguides using nanochannel-guided lithography (NCL) [80]. This technique enabled the continuous extrusion of a polymer to form the waveguide’s core ridge directly onto a polymer undercladding layer. A precisely cleaved mold edge with microtrench patterns was used to slide steadily over a UV-curable resin-coated substrate under conformal contact. The resin and substrate materials were selected to meet the optical requirements for the waveguide core and cladding. Localized heating of the mold allowed for control over the resin’s viscosity, ensuring optimal filling of the microchannels and smooth extrusion, which was then cured using UV light. The extruded polymer core exhibited a highly smooth surface, advantageous for low-loss optical waveguiding—an effect that was confirmed through insertion loss measurements. This approach provided a practical and scalable route for the continuous fabrication of waveguides and integrated photonic components [80].

Ormocore and Ormoclad are UV-curable hybrid polymer materials supplied by Micro Resist Technology GmbH (Berlin, Germany), widely used in integrated photonic devices [86]. Ormocore, with a refractive index of ~1.555, serves as the core material, while Ormoclad, with a slightly lower refractive index (~1.537), is used as the cladding. Both are based on ORMOCER^®^ technology, combining inorganic siloxane networks with organic methacrylate groups, offering excellent optical transparency, mechanical stability, and compatibility with standard lithographic and imprinting techniques. These materials are particularly suited for fabricating low-loss optical waveguides in the visible to NIR range.

**Figure 1:**
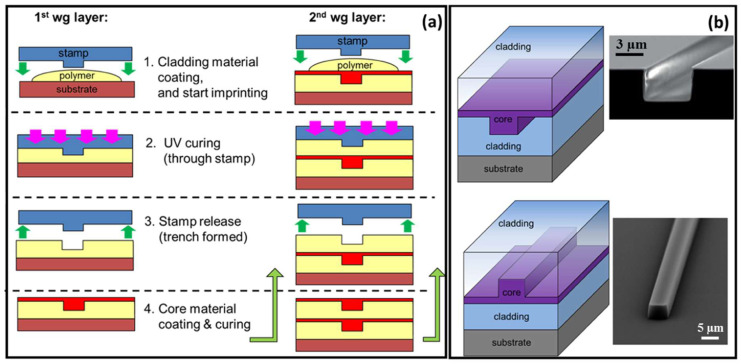
(**a**) Sequential fabrication steps for multilayer inverted rib waveguides using UV-NIL, (**b**) Illustrations and SEM images depicting the inverted rib waveguide process (top) and the rib waveguide process (bottom) [87].

UV-NIL was employed to fabricate single-mode polymer waveguides for both single- and multilayer passive optical interconnects [87]. The NIL process primarily involves two stages: the creation of the stamp and the NIL itself. The inverted rib waveguide process, illustrated in Figure 1a, starts with spin-coating a 30 µm thick layer of a lower-index cladding material, Ormoclad, to provide optical isolation between the waveguide mode and the substrate (Figure 1(a1)). An imprint stamp is then brought into contact with this cladding layer to form trench waveguides, followed by UV curing (Figure 1(a3)). A typical trench structure is shown in Figure 1b (top-right). These trenches are filled with a higher refractive index material, a mixture of Ormocore and Ormoclad, applied via spin-coating to create a slab layer that covers the trenches completely (Figure 1(a4)). It is essential that this layer is smooth and fills the trenches. After curing the core, a final spin-coating and curing step of Ormoclad, the lower-index material, is performed on top, completing the fabrication of the single-level optical waveguide [87].

The fabrication tolerances associated with imprint lithography were thoroughly investigated, and an experimental approach was developed to quantify subtle variations in the refractive index contrast between the core and cladding of the fabricated structures. Demonstrations included 1 × 2 optical splitters, realized through directional couplers and multimode interference (MMI) devices, which achieved insertion losses below 0.45 dB and exhibited minimal output power imbalance, measured at 0.02 ± 0.01 dB. Furthermore, an optical via was fabricated to facilitate vertical signal transfer between optical layers, maintaining an insertion loss of less than 0.45 dB. A 1 × 4 two-dimensional optical port was also demonstrated, successfully dividing the input signal among four outputs with a measured insertion loss of 1.2 dB [87].

### 3.2. Soft Lithography

Soft lithography, including microcontact printing, is a low-cost, versatile technique that uses elastomeric stamps commonly made of polydimethylsiloxane (PDMS) to transfer patterns onto substrates [88]. The stamps are molded from a master template and can conform to both flat and curved surfaces, enabling patterning on non-planar substrates [89]. Soft lithography is especially attractive for patterning organic materials, biomolecules, and for applications such as microfluidics and flexible electronics. Typical resolution is around 50 nm, although achieving features smaller than 30 nm can be challenging due to stamp deformation and ink diffusion [90,91]. While inexpensive and easy to implement, soft lithography is less suited for producing very high-density or high aspect-ratio structures compared to NIL [27].

Soft lithography replica molding was used to fabricate microring resonator filters composed of unclad polystyrene (PS) and clad SU-8 [92]. The PS resonator was demonstrated to possess an intrinsic quality factor of 1.0 × 10^4^ at a wavelength of 1.55 μm, whereas the SU-8 resonator has shown a quality factor of 7100. Extinction ratios of −12 dB for the PS filter and −20 dB for the SU-8 filter were measured, with net insertion losses of 6.7 dB and 9.9 dB, respectively. These findings, characterized by high quality factors and significant extinction ratios, have confirmed the practicality of soft lithography replica molding for the fabrication of integrated optical devices [92].

**Figure 2:**
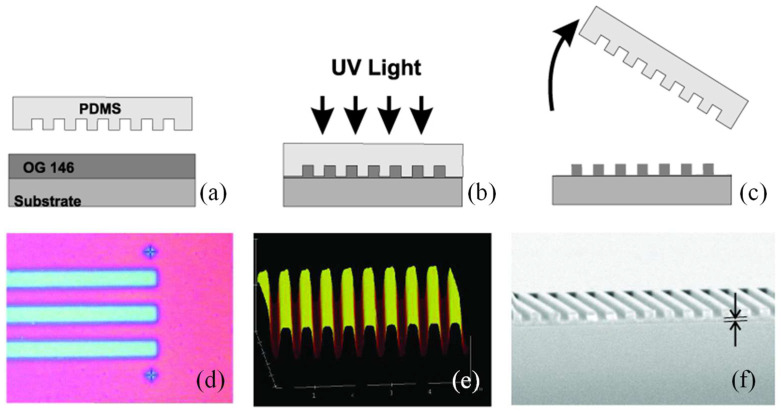
Process flow for transferring the grating pattern: First, a pre-polymer is spin-coated onto the wafer (**a**). Next, a PDMS stamp is carefully placed onto the coated layer and exposed to UV light to cure the polymer (**b**). After curing, the elastomeric stamp is peeled away from the wafer, transferring the pattern (**c**). The patterned area is then examined under an optical microscope (**d**), followed by detailed surface imaging using atomic force microscopy (AFM) (**e**). Finally, scanning electron microscopy (SEM) provides a close-up view of the grating structure, including the residual layer (**f**) [27].

A polymeric waveguide Bragg grating filter was produced using a soft lithography method [92]. The master grating pattern was created through electron beam lithography. Through capillary forces and an elastomeric stamp, consistent grating patterns featuring ultra-thin residual layers were transferred onto a UV-curable polymer, eliminating the need for an imprinting device. The waveguide layer, made from BCB optical polymer, was formed via standard optical lithography. The grating transfer process is illustrated in Figure 2a–c. The master grating was patterned on a silicon wafer using e-beam lithography and then etched by reactive ion etching. The groove dimensions are as follows: 515 nm period, 200 nm depth, 100 μm width, 2 cm length, and 0.5 duty cycle. The elastomeric stamp was made by casting PDMS over the master and curing at 75 °C for 2 h before peeling it off. A 5 mm thick PDMS layer sufficed for replication, although composite stamps can enhance pattern fidelity [11].

The PDMS stamp was used to imprint the grating onto a low-viscosity UV-curable pre-polymer (OG 146). The pre-polymer was spin-coated (~1 μm thick) on a silicon wafer with a 7 μm oxide layer. The PDMS stamp was placed gently onto the polymer, allowing capillary action to fill its grooves. UV exposure for 5 min cured the polymer, after which the stamp was removed, leaving the grating on the substrate. To evaluate uniformity and residual layer thickness, the pattern was transferred to a silicon wafer. Figure 2d shows a uniform optical image of the grating. The SEM cross-section in Figure 2f reveals a groove depth of ~200 nm and a residual layer thickness of ~100 nm, which depends on the initial polymer thickness. Figure 2e shows the AFM image confirming the 200 nm depth. Thermal contraction of PDMS during curing causes a 1–3% reduction in grating period, adjustable by curing temperature. This shrinkage allows fine tuning of the grating period by up to ~10 nm, shifting the Bragg resonance wavelength by about 30 nm. This technique offered a straightforward and efficient process for fabricating Bragg grating filters [27].

### 3.3. Roll-to-Roll (R2R) Processing

R2R is an industrial-scale fabrication method that excels in producing large-area devices on flexible substrates at very high throughput [25,93]. In R2R processing, a flexible substrate is continuously fed from a roll through various stations that perform coating, printing, embossing, or curing steps. This technique is widely used for manufacturing flexible displays, organic photovoltaics, and disposable sensors [94]. Although the achievable resolution is generally limited to about 100 nm or larger, unless combined with techniques like nanoimprinting, R2R’s key advantage lies in its ability to produce enormous areas rapidly and cost-effectively. The main technical challenges involve precise control of web tension, alignment, and layer registration during processing [95].

The sequential dewetting mechanism was investigated, and a continuous fabrication method for polymeric microstencils was developed using an R2R imprinting system [96]. To enable residual-free patterning via dewetting, the mold material, polymer resin, and substrate were selected based on interfacial surface energy analysis. A paraffin-coated film was chosen as the substrate, as its low surface energy allowed efficient dewetting, while providing stronger adhesion than the PDMS mold, facilitating smooth, continuous demolding during the R2R process.

**Figure 3:**
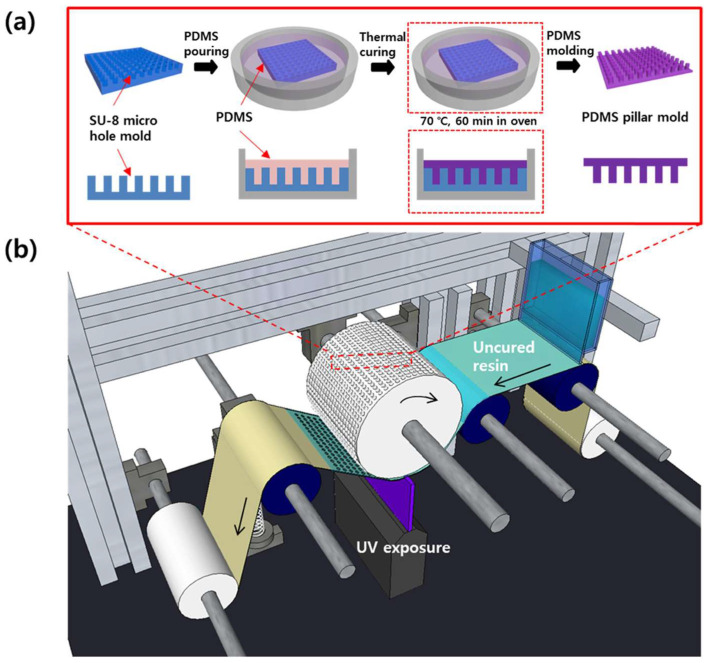
Diagram of the R2R system designed for microstencil production. (**a**) Process flow for preparing the PDMS mold; (**b**) configuration layout of the R2R apparatus used in continuous fabrication [96].

Figure 3 presents a schematic overview of the continuous fabrication process for flexible microstencils using an R2R UV imprint lithography system. The process began with the fabrication of a soft mold embedded with a periodic pillar array, which was produced through a straightforward molding technique (Figure 3a). This flexible mold was subsequently wrapped around a rotating roller, forming the core of the R2R system used to transfer micro-patterns onto a UV-curable resin layer coated on a flexible substrate (Figure 3b). As the roller advanced, the mold came into conformal contact with the resin-coated substrate, enabling pattern transfer. By tailoring the interfacial surface energies among the mold, resin, and substrate, localized dewetting of the liquid resin was triggered. This occurred specifically at locations where the mold’s pillar tips compressed the resin, thinning the layer enough to expose the underlying substrate. Following UV exposure, the patterned resin layer was cured. The resulting microstencil, featuring well-defined perforations, was then carefully separated from the mold and peeled off the substrate, thereby completing the continuous fabrication process.

Key process parameters, including imprinting speed, mold aspect ratio, and applied pressure, were experimentally varied, and their effects on microstencil geometry were evaluated. Optimal conditions were identified under which microstencils were fabricated continuously with high yield and feature resolution reaching 20 μm in diameter. The method developed was validated as a versatile, scalable, and material-specific approach for high-throughput production of microstencil structures suited to flexible and mass-production applications [96].

### 3.4. Inkjet Printing and 3D Printing

Inkjet printing and 3D printing represent additive, mask-less fabrication approaches that are increasingly being explored for photonic device production [97]. Inkjet printing offers flexibility in pattern design, minimal material waste, and low startup costs, as no physical masks or molds are required. Entry-level research inkjet systems typically cost between USD 5000 and USD 20,000, while more advanced systems can exceed USD 150,000, depending on resolution and material compatibility. Material costs, such as nanoparticle-based inks, range from USD 100 to over USD 1000 per liter. It is well-suited for rapid prototyping and custom components. Three-dimensional printing, including advanced techniques like two-photon polymerization, allows for the fabrication of complex three-dimensional photonic structures with feature sizes down to the sub-micrometer or even tens-of-nanometer scale [98]. However, such high-resolution systems, such as two-photon lithography platforms, can range from USD 200,000 to over USD 1 million, and require specialized photoresist materials costing USD 300 to USD 1000 per milliliter. While 3D printing offers superior resolution, it is generally slow and less suited for large-area production. Additionally, issues such as ink formulation, nozzle clogging, and surface roughness must be addressed to ensure consistent, high-quality results [99].

In terms of comparative performance, NIL offers the highest resolution (<10 nm) at moderate cost, particularly when mold costs can be amortized over large production volumes [100]. Soft lithography provides lower resolution (typically ~50 nm) but at very low cost and with greater substrate versatility [91]. R2R processing delivers unmatched throughput and the lowest per-area cost, but at the expense of resolution, unless integrated with higher-resolution patterning techniques [101]. Inkjet and 3D printing provide design flexibility and low setup costs but generally lag in throughput and resolution for large-area fabrication. The choice among these techniques depends heavily on the specific requirements of resolution, scale, cost, and substrate compatibility for the intended application [102].

Techniques like inkjet, electrohydrodynamic, and aerosol printing, classified as noncontact methods, have garnered significant interest for their ability to precisely deposit functional materials essential for applications in printed electronics, photonics, biotechnology, microfluidics, and optoelectronics. Theiler et al. presented a method for fabricating tapered optical waveguides via printing, achieving propagation losses as low as 0.19 dB/cm, with an average of 0.61 ± 0.26 dB/cm [103]. Continuous structural features are critical for ensuring functionality in printed designs, yet they are frequently disrupted by capillary effects. An inkjet printing approach was utilized that harnesses these capillary forces to form liquid bridges, guiding them into continuous, smooth lines even on substrates with varying wettability [103].

**Figure 4:**
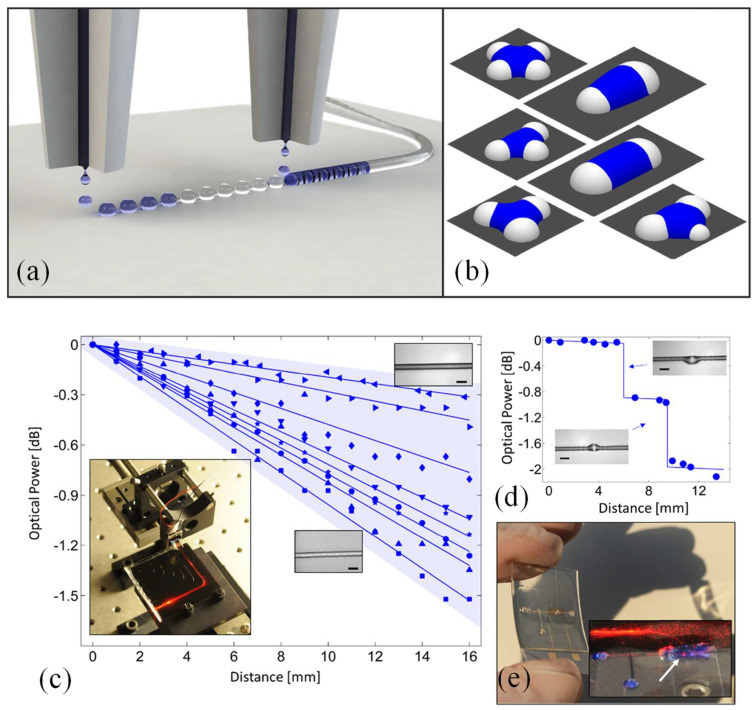
Overview of the printing technique. (**a**) Sequential droplet placement results in the formation of self-aligned liquid bridges, enabling the construction of complex structures. (**b**) Examples of capillary bridges (shown in blue) formed between two or more anchoring caps (shown in white), with surface profiles generated using the Surface Evolver software. (**c**) Attenuation of 650 nm light in straight, printed waveguides. (**d**) Loss across waveguides with induced defects. Insets highlight bulges causing ~0.8 dB scattering loss. (**e**) Fully printed chip integrating optical, electrical, and fluidic components on flexible film. Inset: red laser light outcoupled into a blue sucrose-filled microchannel (arrow) [103].

Figure 4a outlines the fabrication strategy. The process begins with the deposition and stabilization of discrete spherical caps, which act as anchoring sites. When droplets are subsequently introduced between these caps, capillary forces drive the formation of self-aligned liquid bridges. These bridges naturally align into straight paths and can then be solidified to form smooth printed lines. Achieving optimal results requires that fresh ink properly wets the previously deposited caps. To ensure isolation between bridges and prevent bulging, the volume of each bridge must be carefully controlled relative to the spacing between caps. Each bridge should contact no more than half of each adjacent cap, allowing distinct bridges to form without fluid transfer between them. Under these conditions, the behavior of individual bridges can be analyzed independently, without compromising the accuracy of the physical model. Importantly, capillary bridges are not restricted to forming between two identical caps. Configurations involving three or more caps can be used to construct more complex geometries, such as junctions and sharp turns. As illustrated in Figure 4b, simulated liquid bridges generated using Surface Evolver demonstrate a variety of forms that can serve as primary structural elements in printed patterns.

Figure 4c illustrates the attenuation of optical power along straight, defect-free printed waveguides. To assess optical losses after the bend section, the sliding prism technique was employed (see setup photograph in the inset of Figure 4c; the curved configuration prevents direct laser exposure to the sensor). By incorporating measures such as substrate blackening, masking of adjacent waveguides and other scattering sources, and performing background correction (via reference measurements without the prism), the light output was accurately recorded using a photodiode. These measurements, conducted at a wavelength of 650 nm, revealed an average optical loss of 0.61 ± 0.26 dB/cm across ten samples with cladding layers thicker than the optical wavelength, regardless of whether PET or glass was used as the substrate. In optimal conditions, where waveguides were particularly smooth (see top-right inset in Figure 4c), losses as low as 0.19 dB/cm were observed. Such performance is comparable to conventional fabrication methods like photolithography or reactive ion etching. Surface quality was found to be a critical factor: for instance, even slight surface roughness could increase losses to around 0.8 dB/cm (see middle inset of Figure 4c).

Figure 4d demonstrates the impact of structural defects on light transmission. Waveguides containing intentionally introduced bulging irregularities showed a minimum of 0.8 dB loss per defect. These findings emphasize the importance of producing optically smooth structures, which is a feature that the presented capillary-bridge-based printing technique can consistently achieve. This printing strategy is not confined to optics alone. By selecting different ink materials, the method can be extended to fabricate devices with a wide range of functions. It opens the door to cost-effective, customizable, and fully integrated optical systems produced via additive manufacturing on a single platform. As shown in Figure 4e, the versatility of this approach is exemplified by a fully printed prototype comprising a microfluidic channel, a tapered optical waveguide (with light visibly coupled into the blue-dyed fluid), and a metallic comb capacitor. This integration demonstrates the method’s promise for creating multifunctional, flexible devices even on delicate or uneven surfaces [103].

**Table 2:** Characteristic comparison of NIL, soft lithography, R2R processing, inkjet printing, and 3D printing for the fabrication of polymer sensing devices.

Technique	Resolution	Throughput/Scalability	Cost	Material Compatibility	Design Flexibility	Typical Use in Polymer Sensors
Nanoimprint Lithography (NIL) [104]	High (sub-10 nm possible)	Moderate (batch or step-and-repeat; scalable via R2R NIL)	Moderate (expensive tooling but low per-unit cost)	Broad (polymers, composites, functional layers)	Low (pattern fixed by mold)	High-resolution features for nanoscale sensing structures
Soft Lithography [105]	High (100 nm–1 µm typical)	Low to moderate (lab scale, small batch production)	Low to moderate (low tooling cost, manual steps)	Wide (elastomers, hydrogels, various polymers)	Moderate (mold defines pattern, but flexible fabrication)	Microfluidics, flexible sensor substrates, surface patterns
Roll-to-Roll (R2R) [106]	Moderate (10 µm–100 µm typical)	Very high (continuous, large-area production)	Low (low cost per unit at scale)	Mostly flexible substrates (PET, PEN, flexible polymers)	Low to moderate (depends on integrated patterning tech)	Large-area flexible and wearable sensors
Inkjet Printing [107]	Moderate (20 µm–50 µm typical)	Moderate (depends on printer speed)	Low (digital, no masks required)	Solution-processable polymers, functional inks	High (digital patterning, easy to modify designs)	Patterned electrodes, sensing layers, functional coatings
3D Printing (e.g., extrusion, photopolymerization) [108]	Low to moderate (10 µm–100 µm, depending on method)	Low to moderate (slow compared to R2R)	Low to moderate (depends on printer type)	Limited (depends on printable polymer and ink formulation)	Very high (complex 3D geometries possible)	Prototyping, custom sensor housings, integrated structures

## 4. Polymer Photonic Structures for Sensing

The sensing capabilities of polymer photonic devices are fundamentally defined by the optical structures that guide and manipulate light within the sensor. These architectures enable precise control over light–matter interactions, translating external physical, chemical, or biological changes into detectable optical signals. This section reviews the principal photonic structures employed in polymer-based sensors, each offering distinct advantages in terms of sensitivity, compactness, and fabrication compatibility. Planar, rib, and slot waveguides (Section 4.1) leverage strong evanescent fields for efficient refractive index sensing. Bragg gratings (Section 4.2) provide narrowband spectral responses suitable for temperature, pressure, and chemical detection. Photonic crystal slabs (Section 4.3) utilize bandgap effects and resonant cavities to enhance analyte interaction. Ring and disk resonators (Section 4.4) offer compact, high-Q platforms ideal for detecting subtle environmental changes. Lastly, interferometric configurations such as Mach–Zehnder interferometers (Section 4.5) deliver high-precision phase-based sensing. Each structure is uniquely enabled by the material and processing advantages of polymers, facilitating integration into flexible, low-cost, and scalable sensing platforms. The following subsections detail the operating principles, fabrication techniques, and sensing performance of these key architectures.

### 4.1. Waveguides (Planar, Rib, Slot)

Planar and rib polymer waveguides provide a compact platform with strong evanescent fields beneficial for sensing refractive-index changes [109,110]. Polymers like SU-8, BCB, PMMA, and Cytop enable easy fabrication via UV imprinting, hot embossing, or R2R nanoimprinting, offering low cost, biocompatibility, and tunable thermo-optic properties for temperature and biomarker detection [111]. For example, SU-8 and BCB waveguides in Mach–Zehnder configurations have been used for real-time clinical assays and environmental monitoring [112]. Slot waveguides offer strong potential for optical sensing due to their ability to concentrate light in low-index regions, enhancing analyte interaction [113]. Bettotti et al. showed that sensitivity in such structures depends more on careful design than on index contrast alone [114]. This enables high-performance sensing even with low-index materials like polymers. Polymers further provide advantages in cost, functionalization, and fabrication, while remaining compatible with CMOS processes and integrated photonic circuits. Low-index slot waveguides are promising as compact alternatives to fiber capillary sensors and in ring resonator designs. These findings align with the capabilities of established polymer photonics technologies [114].

**Figure 5:**
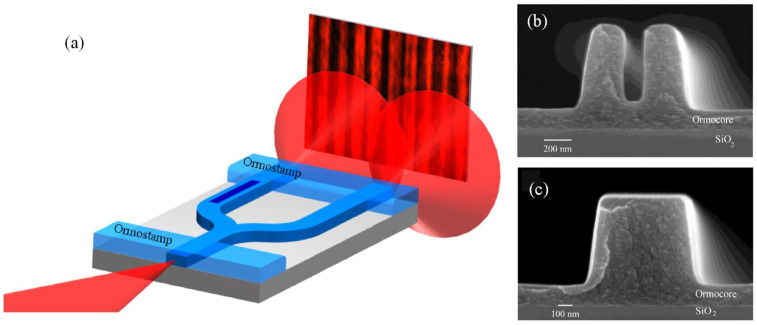
(**a**) Schematic illustration of the slot-based Young interferometer and the experimental measurement setup. The left arm features a slot waveguide section that introduces a phase difference between the two paths, while the right arm serves as a reference using a standard ridge waveguide. SEM cross-sectional images of (**b**) the slot waveguide used for sensing and (**c**) the reference ridge waveguide [113].

A flexible, low-cost CO_2_ sensor was demonstrated using PMMA planar waveguides coated with a uniform ZIF-8 film [43]. Fabricated by hot embossing and simple solution processing, the device achieved ≈2.5 μW/5 vol% sensitivity, rapid ~6 s response, and excellent adsorption–desorption reversibility offering a scalable platform for on-chip gas sensing. In another work, a highly sensitive refractive index sensor was designed and implemented using a Young interferometer integrated with a slot waveguide structure (Figure 5a) [115]. The sensor was fabricated on a polymer-based platform through a simple molding process and operated at a visible wavelength of 633 nm. The SEM image of the slot and standard ridge waveguide is shown in Figure 5b and Figure 5c, respectively.

To evaluate its performance, phase shifts in the interference pattern were measured using glucose-water solutions of varying concentrations under both TE and TM polarization modes. Experimental results showed that the sensor could detect refractive index changes as small as 6.4 × 10^−6^ RIU. Moreover, the interferometric design exhibited inherent compensation for temperature-induced variations. Simulations indicate that the design with both arms exposed is less than half as sensitive to temperature variations compared to the configuration with a shielded reference arm. This demonstrates that the proposed geometry effectively mitigates noise resulting from temperature changes. These results confirm the potential of polymeric slot waveguide interferometers as a cost-effective and efficient solution for high-performance refractive index sensing [115].

### 4.2. Bragg Gratings

Planar and fiber Bragg gratings made of polymers serve as high-resolution, label-free sensors [116]. Polymer optical fiber Bragg gratings (FBGs) allow multipoint sensing under WDM regimes and deployment outside labs, e.g., humidity, strain, or temperature sensing via thermal annealing shifts [117]. On-chip surface-relief Bragg gratings in photoresists (like IP-Dip) are produced via 3D laser lithography and complex imprinting, enabling reflection at ~1543 nm and direct fiber coupling [118]. Additionally, planar waveguide Bragg gratings using imprint or hybrid polymer glass structures detect temperature and humidity with good sensitivity [119]. Grating designs such as apodized, chirped, and multimode planar have been analyzed for realistic sensor deployment [120,121,122].

Li et al. reported a low-cost polymer optical waveguide pressure sensor featuring an integrated Bragg grating [31]. The device exploited the polymer’s low Young’s modulus for high sensitivity, achieving 1.275 nm/kPa over a 0–12 kPa range with 1 kPa accuracy. It responded reliably to pulse signals, showing strong promise for applications in blood pressure monitoring, sleep analysis, and tactile sensing [31].

**Figure 6:**
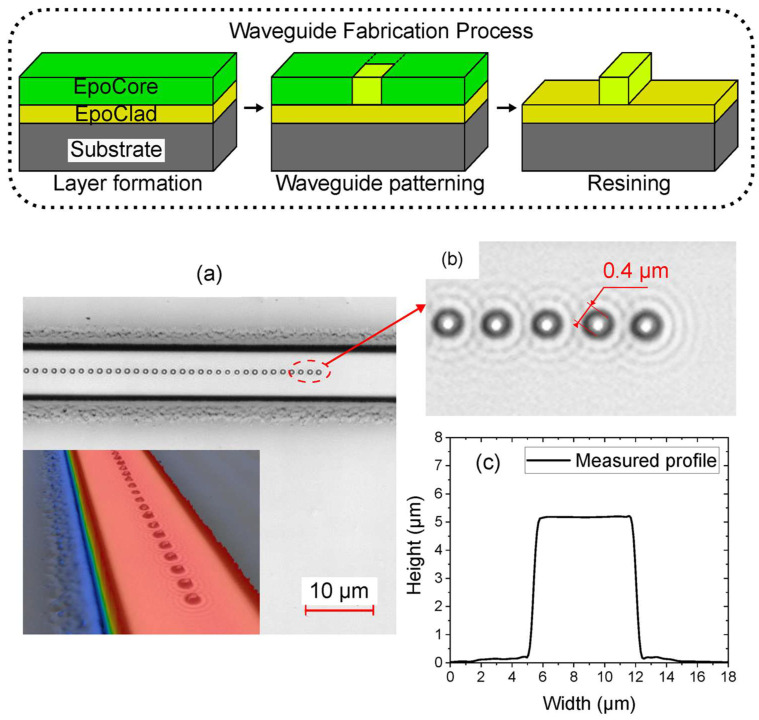
Illustration (dashed box) outlining the waveguide fabrication workflow along with an image of the final structure obtained via laser scanning microscopy (LSM). (**a**) Microscope image of the ridge waveguide with Bragg grating (RWBG), with a 3D height map shown in the inset. (**b**) Magnified view highlighting the laser-written Bragg grating pattern. (**c**) Cross-sectional profile of the polymer ridge waveguide [123].

Recently, an integrated optical sensor based on an evanescent Bragg grating was developed for chemical sensing applications [123]. The sensor design combined a few-mode planar polymer waveguide with microchannel structures, both fabricated from epoxide-based polymers. The Bragg grating was introduced into the waveguide using a point-by-point direct inscription method. Figure 6 highlights the fabrication process in the region outlined by dashed lines. The waveguide, observed at 100× magnification, and its cross-sectional profile measured using a laser scanning microscope are shown in Figure 6c. The cross-section indicates that the waveguide measures approximately 6.5 µm in width and 5 µm in height. Figure 6a presents a laser scanning microscope (LSM) image of the Bragg grating structure. The inset provides a 3D surface profile of the waveguide with the laser-written Bragg grating, also captured by LSM. In Figure 6b, a top-view image reveals the refractive index modulation points produced by individual laser pulses. These grating points, created using a 20× focusing objective, have an average diameter of about 0.4 µm. The overall length of the Bragg grating extends 3 mm along the waveguide.

The sensor’s performance was evaluated through temperature and chemical sensing experiments. Temperature sensitivity was assessed by monitoring the shift in the central wavelength of the reflected Bragg signal, yielding a sensitivity of −47.75 pm/K. To demonstrate the versatility of the evanescent field interaction, two application cases were investigated. First, in refractive index sensing, the device achieved a sensitivity of 6.5 nm/RIU when tested with various aqueous solutions ranging from air to nearly pure isopropyl alcohol (99.9%). Second, for gas-phase hydrogen detection, the waveguide was coated with a functional layer of palladium nanoparticles, enabling reliable detection of hydrogen concentrations up to 4 vol%. The results underline the strong potential of this Bragg grating-integrated waveguide sensor for use in compact lab-on-a-chip systems, particularly for applications requiring simultaneous temperature monitoring and chemical detection [123].

### 4.3. Photonic Crystal Slabs

Photonic crystal (PhC) slab sensors harness slow-light effects and cavity modes to enhance analyte interaction [124]. Hermannsson et al. introduced a disposable PhC slab sensor made entirely from polymer-based materials, optimized for refractive index and biological sensing [125]. The device demonstrates a sharp resonance response, with a full width at half maximum (FWHM) of less than 1 nm, and achieves a sensitivity of 31 nm per refractive index unit (RIU) in environments with refractive indices near that of water. When combined with a spectrometer offering a resolution of 12 pm/pixel, the sensor is capable of detecting changes as small as 4.5 × 10^−6^ RIU. Structurally, the sensor comprises two distinct layers: a base layer of low-refractive-index polymer featuring periodic surface modulation, and a top layer of high-index inorganic–organic hybrid polymer, uniformly coated and doped with zirconia (ZrO_2_) nanoparticles. The fabrication process employs cost-efficient, vacuum-free methods, specifically UV nanoimprinting and spin-coating, making the sensor suitable for scalable production and single-use applications [125].

**Figure 7:**
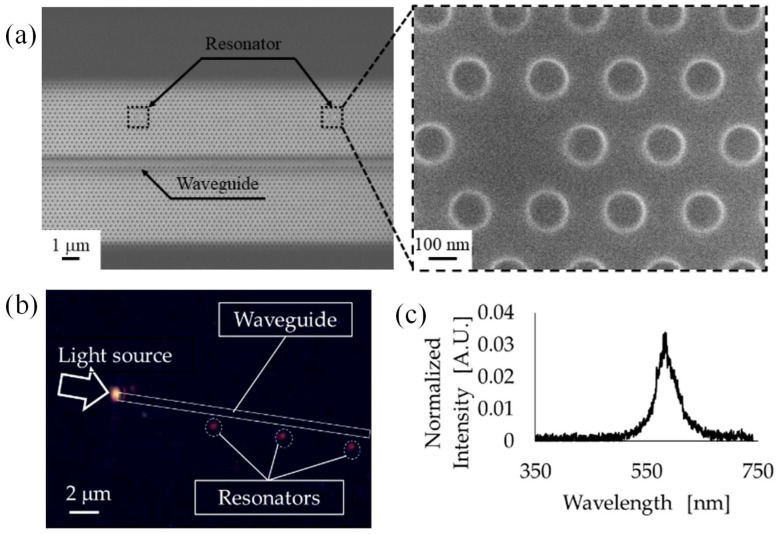
(**a**) SEM image showing the polymer PhC resonator integrated with a waveguide structure. (**b**) Optical microscope image highlighting the PhC resonator and waveguide structure. (**c**) Corresponding light spectrum measured at the resonator [126].

Sun et al. presented a highly sensitive optical sensing platform based on a polymer PhC resonator integrated with a waveguide and designed with cavities of multiple sizes [126]. The structure’s optical properties were investigated using finite-difference time-domain (FDTD) simulations. For fabrication, electron beam lithography was employed to pattern the PhC and waveguide on a photoresist layer deposited over a gold-coated silicon substrate. SEM image of the polymer PhC resonator integrated with a waveguide structure is shown in Figure 7a. The sensing mechanism relies on monitoring resonant light that was guided through the waveguide and confined within the PhC resonator. To experimentally validate the sensor’s response, a layer-by-layer (LbL) deposition technique was used to apply successive polymer layers. This deposition induced measurable shifts in the resonant wavelength, which were tracked in real time. An optimized design featuring a long cavity formed by introducing a three-hole defect demonstrated a resonance peak shift of 5.26 nm per deposited layer. In the dark-field image presented in Figure 7b, resonant light was distinctly visible at the location of the resonator. The corresponding spectrum in Figure 7c confirms a resonance peak at 584 nm with a full width at half maximum (FWHM) of 48 nm. These results indicate that the polymer-based PhC resonator, in conjunction with the waveguide structure, provides an effective and scalable approach for refractive index and thin-film sensing applications [126].

### 4.4. Ring/Disk Resonators

Polymer-based microring/disk resonators offer compact high-Q sensing: polymers like SU-8, BCB via imprinting or lithography achieve Q ≳ 10^4^ and support detection of proteins, temperature shifts, or gases [127,128,129,130]. Madani et al. presented the design, fabrication, and characterization of a polymer-based microring resonator using advanced electro-optic materials [129]. The device, structured in an add/drop configuration, was fabricated using laser beam direct-write lithography with 1 μm resolution. Ormocore and Ormoclad polymers were chosen for their low optical losses at 1300 nm and 1550 nm. Characterization at 1550 nm was carried out using tapered fibers for light coupling and an optical spectrum analyzer for signal monitoring. The fabricated resonator exhibited a 0.3 nm bandwidth, 0.8 nm free spectral range, and a finesse of 2.6 [129].

Zhang et al. introduced an advanced ultrasonic detector that offers both exceptionally broad bandwidth and high sensitivity [131]. This device was based on an imprinted polymer optical microring and demonstrates an acoustic response extending up to 350 megahertz at the three-decibel point. It achieved a noise-limited minimum detectable pressure as low as 10^5^ pascals within this frequency range. When employed in photoacoustic imaging, the detector provides significantly improved axial resolution compared to conventional ultrasound detectors. It achieved an axial resolution below three micrometers, representing more than a twofold improvement over the previously reported best performance. The miniature cavity height of the microring ensured a wide frequency response, while the high optical quality factor enhances detection sensitivity. This work demonstrated that polymer-based miniature microring resonators served as high-performance ultrasonic detectors with strong potential for generating volumetric photoacoustic images at cellular and subcellular resolution in three dimensions [131].

**Figure 8:**
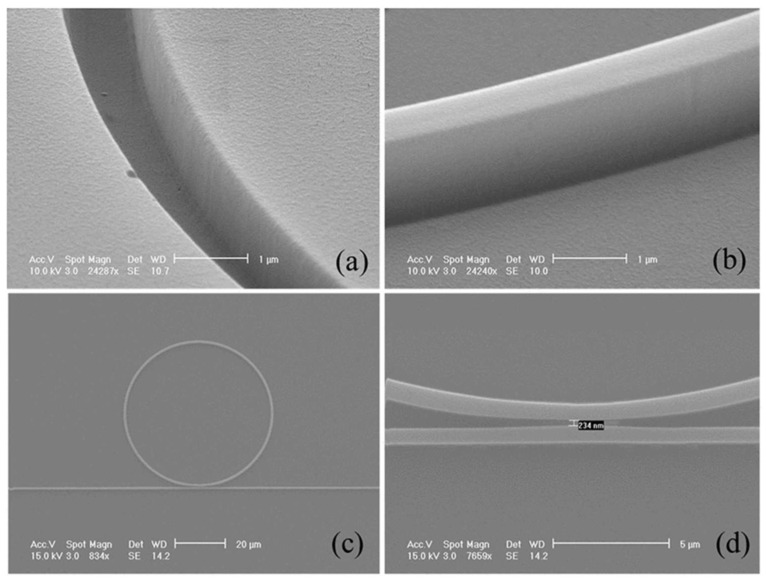
SEM images showing the mold and fabricated polymer microring resonator: (**a**) sidewall structure of the SSQ mold, (**b**) sidewall profile of the SU-8 microring, (**c**) full view of the microring structure, and (**d**) close-up of the coupling gap between the microring and the adjacent bus waveguide [132].

In another study, an ultrahigh-Q polymer microring resonator was systematically studied for its potential in biosensing applications [132]. By refining both the device architecture and fabrication method, the resonator was engineered to operate in a slightly under-coupled regime, achieving a record intrinsic Q factor of 8.0 × 10^5^. This high-Q operation enables enhanced sensitivity and a low detection threshold. Figure 8 presents SEM images of both the mold and the fabricated polymer microring resonator. Images (a) and (b) reveal that the sidewalls of the SSQ mold and SU-8 microring are smooth and well-defined. The top-view of the complete microring structure is shown in (c), while the zoomed-in image in (d) confirms a coupling gap of 234 nm, consistent with the intended design specifications. Experimental evaluation using bovine serum albumin demonstrated successful detection of a surface mass density as low as 12.7 pg/mm^2^ through physical adsorption. The system exhibited noise-equivalent detection limits of approximately 5.3 pg/mm^2^ via wavelength shift measurements and 55.9 fg/mm^2^ using intensity-based detection. These results highlight the exceptional sensing capabilities of polymer microring resonators when optimized for on-chip biosensing [132].

### 4.5. Interferometric Configurations (e.g., Mach–Zehnder)

Mach–Zehnder Interferometers (MZIs) in polymer photonics excel in measuring small phase shifts via evanescent–field interactions [133,134,135,136,137]. Xiao et al. presented all-polymer MZIs designed for environmental sensing and fabricated via large-area printing for low-cost production [133]. Unlike conventional MZIs that require complex processing of an interaction window, this design uses an asymmetric structure to avoid such steps, making it suitable for polymer fabrication. Optimized design criteria were developed to enhance sensitivity and performance. Experimental tests with a chemical sensor demonstrate the effectiveness of this approach [133].

**Figure 9:**
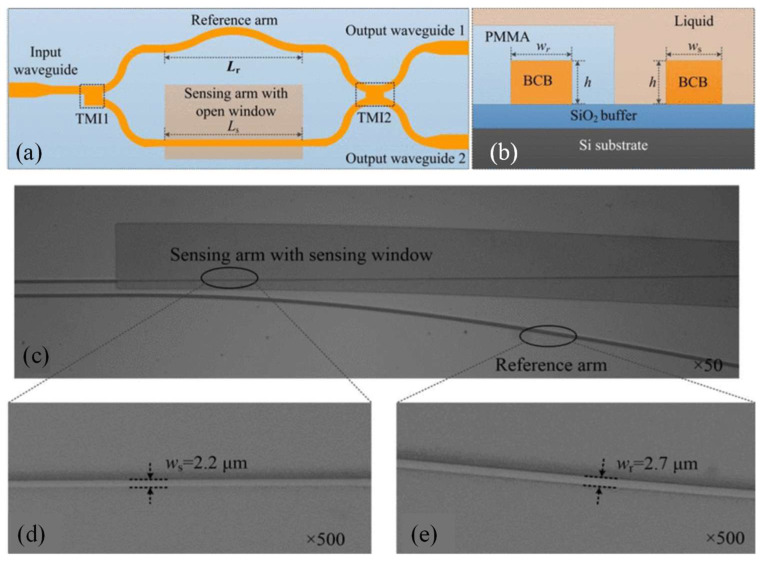
(**a**) Schematic and (**b**) cross-sectional views of the two arms in the proposed MZI sensor. Optical microscope images of (**c**) the sensing and reference arms, (**d**) sensing waveguide, and (**e**) reference waveguide [135].

Temperature sensors capable of covering broad measurement ranges are essential for various industrial applications. Liu et al. introduced a polymer-based asymmetric MZI designed for temperature sensing [134]. Experimental data reveal that the interference output varies periodically with temperature changes. The sensor achieves a measurement range of 120 °C and a sensitivity of 0.27 rad/°C. These results demonstrate a promising, cost-effective solution for high-performance temperature sensing over an extended range [134]. Ma et al. introduced a cost-efficient polymer-based MZI sensor for detecting liquid refractive index (Figure 9a) [135]. Figure 9b schematically presents the cross-sectional structure of the two arms of the proposed MZI, including the materials utilized in this study. Figure 9c–e display optical micrographs of key regions of the fabricated MZI captured at various magnifications, along with the measured widths of the waveguide arms. The sensor’s sensitivity is enhanced by optimizing the reference arm length to minimize the optical path difference (OPD) between the interferometer arms. Several devices were fabricated with a constant sensing arm length of 7900 µm but varied reference arm lengths (7900.0, 7942.5, 7950.9, 7962.2, and 7969.5 µm). Among these, the highest RI sensitivity achieved was 33,662.8 nm/RIU, while the detection range remained stable at approximately 0.0041 ± 0.0002 RIU [135].

Using two-photon polymerization (TPP), an on-chip MZI sensor with asymmetric polymer waveguide arms was fabricated [136]. The differing lengths of the interference arms cause variations in their effective refractive indices with temperature changes, resulting in an initial sensitivity of −348.5 pm/°C over a 20 °C to 60 °C range. When coated with the temperature-responsive polymer polydimethylsiloxane (PDMS), the sensor’s sensitivity improved approximately sixfold to around −2.01 nm/°C between 30 °C and 70 °C, maintaining consistent behavior during both heating and cooling cycles. Compact and integrated, this photonic sensor combines high temperature sensitivity, biocompatibility, and stability, making it ideal for precise temperature monitoring in small or complex environments [136].

## 5. Applications

Polymer photonic sensors have emerged as versatile and high-performance platforms for a broad range of sensing applications. Their tunable optical properties, mechanical flexibility, and ease of fabrication make them particularly attractive for use in real-world environments where conventional photonic materials may fall short. This section explores the key application domains where polymer photonic sensors are making significant impact, including environmental monitoring, point-of-care medical diagnostics, food safety, and structural health monitoring.

### 5.1. Environmental Monitoring

Polymer photonic sensors are increasingly used for real-time, in situ monitoring of environmental parameters such as humidity, temperature, gas concentrations, and water quality [21,138,139]. Their optical transduction mechanisms, such as changes in refractive index, fluorescence, or photonic bandgap shifts, enable high sensitivity and selectivity to a wide range of analytes. Functionalized polymer photonic structures have shown excellent responsiveness to gases such as ammonia, NO_2_, and VOCs [20,140]. Colorimetric and photonic crystal-based sensors using polymers like polyaniline and PDMS composites exhibit strong selectivity and visual detectability [141,142]. Hydrogel-based photonic crystals and waveguide sensors enable detection of pH, heavy metal ions (e.g., Pb^2+^, Hg^2+^), and pollutants with fast response times and minimal instrumentation [143,144]. Polymers like polyacrylamide, often doped with ion-specific ligands or dyes, have enabled label-free optical detection in aqueous environments [145].

A polymer-based evanescent Bragg grating sensor was developed by integrating a few-mode planar waveguide into microfluidic channel structures [123]. The waveguide and microchannels were fabricated using epoxide-based polymers, and the Bragg grating was inscribed post-fabrication using a point-by-point direct writing technique. Characterization was performed to assess the sensor’s response to various chemical stimuli. A temperature sensitivity of −47.75 pm/K was obtained by monitoring the central wavelength shift in the reflected Bragg signal. To demonstrate functionality based on evanescent field interactions, two application cases were evaluated. Refractive index sensing was carried out using a range of aqueous solutions, achieving a sensitivity of 6.5 nm/RIU from air to 99.9% isopropyl alcohol. For gas-phase hydrogen detection, the sensor surface was coated with a palladium nanoparticle layer, enabling reliable detection of hydrogen concentrations up to 4 vol%. The results confirmed reproducibility and robustness in both liquid- and gas-phase environments. These findings highlight the strong potential of Bragg grating-integrated polymer waveguide sensors for lab-on-a-chip platforms, especially in compact, multifunctional chemical and environmental monitoring systems [123].

A novel optical waveguide (OWG) sensor for low-humidity detection was proposed, in which a localized polyvinylpyrrolidone (PVP) film was applied [138]. The sensor was fabricated by spin-coating a PVP layer onto a potassium ion-exchanged glass waveguide. A humid air stream with relative humidity ranging from 10.6% to 32% was introduced at room temperature to characterize the sensor’s performance. The surface morphology of the PVP film was analyzed using atomic force microscopy (AFM), and the humidity sensing mechanism was examined through absorption spectral analysis. High sensitivity, stability, and fast response/recovery characteristics were observed during testing. Based on the experimental results, reliable detection of low water vapor concentrations was achieved. As such, the PVP-coated OWG sensor was demonstrated to be a promising and cost-effective candidate for real-time humidity monitoring applications [138].

**Figure 10:**
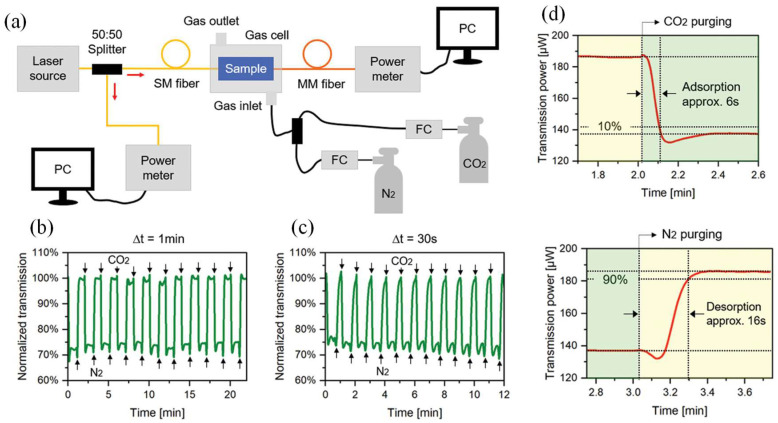
(**a**) Diagram of the experimental arrangement designed to evaluate gas sensing characteristics. Optical signal variation in the ZIF-8-modified PMMA waveguide upon CO_2_ exposure. The response behavior of the metal–organic framework (MOF)-coated waveguide is shown with gas switching intervals of (**b**) 1 min and (**c**) 30 s. (**d**) Response and recovery times during CO_2_ adsorption and desorption processes on the MOF-functionalized waveguide sensor [43].

Recently, a flexible and low-cost gas sensor was developed based on planar polymer optical waveguides coated with a zeolitic imidazolate framework-8 (ZIF-8) thin film for carbon dioxide (CO_2_) detection [43]. The waveguides were fabricated from polymethyl methacrylate (PMMA) through a hot embossing technique, allowing scalable and economical production. A uniform ZIF-8 film was deposited on the waveguide surface using a simple solution-based method, enabling large-scale integration of metal–organic framework materials into sensing devices. Figure 10a presents a schematic representation of the setup implemented for assessing gas sensing performance.

Figure 10b shows the optical transmission of the ZIF-8-coated PMMA waveguide under gas switching intervals of Δt = 1 min. The signal consistently decreased upon CO_2_ exposure and returned during N_2_ purging, demonstrating stable and repeatable sensor performance. To evaluate behavior under faster switching, a second test with Δt = 30 s was conducted (Figure 10c). Although the signal did not fully stabilize during N_2_ purging due to the shorter interval, periodic variation remained clear, confirming the sensor’s high reversibility and reproducibility. The response and recovery times were extracted from the onset to 90% of the steady-state signal (Figure 10d). CO_2_ adsorption and desorption times were approximately 6 s and 16 s, respectively. These fast dynamics result from physisorption within the ZIF-8 layer, enabling rapid gas diffusion and release. The longer desorption time reflects slower gas evacuation but still supports real-time sensing capability. Sensitivity to CO_2_ was measured at approximately 2.5 μW per 5 vol%, with a response time of about 6 s and excellent reversibility in the adsorption and desorption of gas molecules. This sensing approach was demonstrated as a promising solution for the fabrication of reproducible, flexible, and integrated on-chip gas sensors [43].

### 5.2. Point-of-Care Medical Diagnostics

Polymer photonic sensors are well-suited for point-of-care (PoC) diagnostics due to their biocompatibility, low cost, and high sensitivity [20,135]. Interferometric, microring resonator, and photonic crystal slab platforms based on polymers such as SU-8, PMMA, and polycarbonate have enabled biomarker detection at clinically relevant levels.

Examples include optical biosensors detecting glucose, lactate, CRP, and viral antigens (e.g., SARS-CoV-2) using surface-functionalized polymer waveguides and resonators [146]. For instance, a compact, cost-effective, and user-friendly urinalysis device was presented for potential use in home healthcare and clinical environments [146]. The device was designed using a polymeric optical waveguide as the sensor platform, while colorimetric absorption was employed as the sensing mechanism. Quantitative analyses were conducted, and detection limits were achieved below 0.1 g/L for glucose, 0.2 g/L for creatinine, 0.025 g/L for albumin, and 0.1 g/L for total protein. Superior performance over conventional dipstick methods was demonstrated, including enhanced sensitivity, quantification capabilities, and reusability. Further development has been undertaken to miniaturize the system by integrating a light-emitting diode (LED) as the illumination source and a photodiode as the detector. Based on these advancements, the device has been recognized as a promising tool for early diagnosis and routine health monitoring [146].

A polymer optical sensor for detecting effective anti-inflammatory concentrations of peimine was fabricated using a metal-printing technique to define the active waveguide [147]. An erbium-doped copolymer was used as the sensing core, with a grafted PMMA material applied as the cladding. Drug–polymer interactions were analyzed via molecular docking, confirming hydrogen-bonding affinities. An optimized multimode interference (MMI) waveguide structure was designed, and its optical response was simulated for various peimine concentrations. A sensitivity of 2 × 10^3^ RIU^−1^ was achieved, with a resolution of 2.5 × 10^−4^ and a detection limit of 1.3 × 10^−7^ RIU. The sensor effectively detected peimine in the 10–25 mg/L range, showing a 5 dB optical power shift and a 3 dB gain for calibration. This approach was validated as a simple, rapid, and sensitive method for alkaloid drug detection in traditional medicine [147].

Recently, the design, fabrication, and testing of a 3D-printed plasmonic sensor were conducted. A silver–gold bilayer has been deposited onto a polymer waveguide to enhance the surface plasmon resonance (SPR) sensor’s performance [148]. The resulting SPR sensor was realized as a compact, low-cost, and straightforward device. Its sensitivity was demonstrated to be sufficient for biosensing applications by functionalizing the bilayer with specific receptors. The proposed sensing approach was identified as suitable for the production of disposable sensor chips applicable in various fields, including PoC testing and environmental monitoring. Both numerical simulations and experimental validations have shown that the sensor’s sensitivity was improved by using the silver–gold bilayer instead of a gold-only layer [148].

Exceptional sensitivity and selectivity have long been pursued as primary goals in sensor development. To meet these demands, a sensing platform was developed by integrating a molecularly imprinted polymer (MIP) with an optical fiber–waveguide–fiber (OFWF) structure [149]. The OFWF configuration was utilized to efficiently launch and collect light signals, while the MIP layer was designed to provide high molecular recognition capability and sensitivity. In constructing the MIP, 2-phenylphenoxyethyl acrylate, a monomer with a high refractive index, was copolymerized with acrylic acid, a functional monomer. Through this formulation, high-refractive-index MIP layers were fabricated to enable effective extraction of probe light from the waveguide and its transmission to the sensing interface. To enhance analyte permeability, poly(ethylene glycol) 600 diacrylate was employed as a flexible cross-linker, thereby enlarging the polymer mesh size.

Rhodamine B (Rh B), a commonly used dye in textile manufacturing and a potential environmental hazard, was selected as the molecular template. The MIP layer was formed on the waveguide surface through a curing process activated by the evanescent field of 405 nm light propagated within the waveguide. Using this MIP-OFWF system, selective detection of Rh B in the presence of methyl blue was achieved by monitoring their absorption spectra. An ultra-low detection limit of approximately 6.5 × 10^−17^ g/mL was reached, corresponding to an absolute mass detection in the range of 20–30 attograms [149].

**Figure 11:**
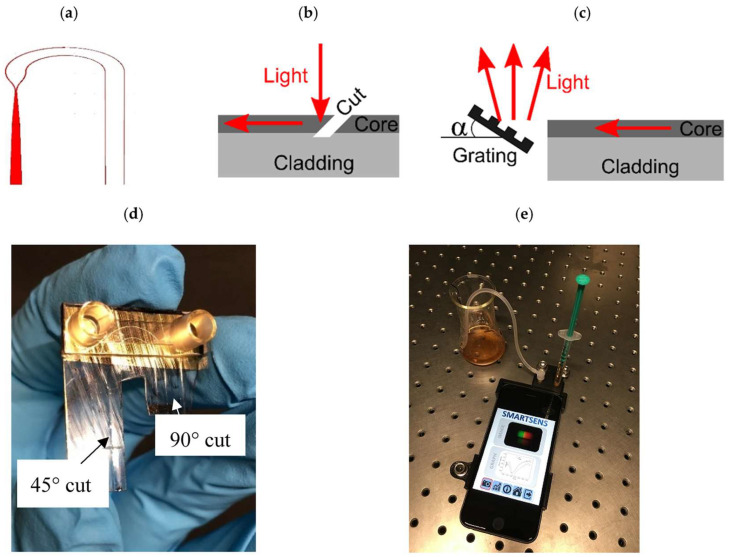
Diagram of the sensor system (**a**) and the light coupling designs for input (**b**) and output (**c**) of the planar-optical waveguide sensor. Input coupling uses a 45° cut for total internal reflection, while output coupling employs a 90° cut and a reflective diffraction grating. After assembling the coupling elements and microfluidic channel (**d**), the sensor chip is enclosed in a 3D-printed housing (**e**) [150].

An all-optical plasmonic sensor platform was developed for smartphone integration, utilizing planar-optical waveguide structures embedded within a polymer chip [150]. The applicability of the system for biosensing was demonstrated through the detection of 25-hydroxyvitamin D (25OHD) in human serum samples, using an aptamer-based assay enhanced with gold nanoparticles (AuNPs). Figure 11a shows the schematic of the planar-optical waveguide structure. The design begins with a broad 1200 µm waveguide segment aligned to the smartphone’s flashlight LED to enhance light coupling. This segment tapers to 200 µm, then splits into two 100 µm wide waveguides forming two separate SPR sensors. Each channel further narrows to 50 µm, matching the dimensions used in previous planar-optical SPR sensor studies. A waveguide bend with a 5 mm radius guides light from the LED to the smartphone camera. The sensor chip was fabricated using the hot embossing technique by Rezem et al. [20,21], imprinting a 25 µm deep waveguide pattern into a 375 µm thick PMMA sheet (refractive index 1.49). The cladding structure was then filled by doctor blading with UV-curable epoxy NOA 63 (refractive index 1.56) and cured under UV light [150].

The planar-optical biosensor chip was mounted in a 3D-printed polymer housing designed to fix the sensor onto an Apple iPhone 6s and enable experiments (Figure 11e). The housing has two parts: one aligns the sensor with the smartphone’s LED and camera, and the other blocks ambient light. Light from the smartphone LED was coupled into the sensor via a 45° cut, creating total internal reflection (Figure 11b). The waveguide ends were cut perpendicularly with a heated blade to direct light onto a reflective diffraction grating, replicated by hot embossing an 1800 lines/mm Thorlabs grating and coated with 100 nm silver (Figure 11c). Positioned at a 7° angle incorporated in the housing, the grating disperses light into the camera, capturing the full LED spectrum. Fluid delivery and waste removal through the microfluidic chip’s Luer adapters were managed using syringes and silicone tubing. The assembled chip and setup are shown in Figure 11d,e [150]. A sensitivity of 0.752 pixels/nM was achieved for 25OHD concentrations ranging from 0 to 100 nM. The waveguide structure was designed to enable both miniaturization and parallelization, allowing for the simultaneous detection of multiple analytes, including biomarkers. The entire optical setup was integrated into a single polymer chip, enabling large-scale and cost-efficient production. The widespread use of smartphone electronics has been considered to make this platform highly promising for lab-on-chip applications [150].

### 5.3. Structural Health Monitoring

In structural health monitoring (SHM), polymer photonic sensors provide robust, lightweight, and flexible solutions for detecting strain, stress, delamination, and cracks [151,152]. Stretchable photonic waveguides fabricated from elastomers like PDMS and polyurethane have been used for distributed strain sensing across complex geometries [53,153]. In recent years, the need for accurate displacement detection in SHM has significantly increased, particularly for applications requiring high-sensitivity sensors capable of detecting displacements from the millimeter to micrometer scale.

Three PDMS-based devices were fabricated and characterized for displacement detection through curvature variation, each embedded with a fiber Bragg grating (FBG) [153]. Three structural designs were employed: a solid device, a perforated device with three central holes, and a notched device featuring three notches. The perforated and notched designs were introduced to allow greater deformation, thereby enhancing sensitivity. A comprehensive evaluation was conducted under various displacement and temperature conditions. Due to the integration of FBGs, which were subjected to tensile or compressive strain depending on the bending direction, a wider measurement range was enabled without compromising sensitivity. Significantly higher sensitivities were observed in the perforated and notched designs when compared to the solid configuration. This enhancement was attributed to the increased stress concentration resulting from structural modifications. All three devices were simultaneously tested under both millimetric and micrometric displacements, and parameters such as curvature sensitivity, displacement sensitivity, temperature responsiveness, hysteresis error, linearity, and repeatability were assessed. The solid device was found to have a sensitivity of 112.7 pm/mm, whereas the perforated and notched devices achieved improved sensitivities of 159.5 pm/mm and 162.7 pm/mm, representing increases of 41.5% and 44.36%, respectively. These outcomes have demonstrated the effectiveness of incorporating geometric modifications in enhancing the performance of PDMS-FBG-based displacement sensors, offering practical advantages for structural monitoring systems [153].

Polymer optical fiber sensors are more widely used in SHM than polymer waveguide sensors due to their superior mechanical flexibility, ease of integration, and cost-effectiveness [6]. Polymer optical fiber sensors offer greater tolerance to bending, stretching, and vibration, making them highly suitable for dynamic and harsh environments typically encountered in civil, aerospace, and mechanical structures [154,155]. Unlike polymer waveguides, which often require precise alignment and are more susceptible to optical losses due to surface imperfections or coupling issues, polymer optical fibers can be easily embedded or surface-mounted without complex fabrication requirements [156]. Additionally, polymer optical fiber sensors are capable of supporting longer sensing distances and multiplexing capabilities, enabling the monitoring of large structures with fewer components [157]. Their resilience, scalability, and simpler installation processes make polymer optical fiber sensors a more practical and reliable choice for widespread SHM applications [158].

The anisotropic nature of fiber-reinforced polymer composites, combined with their vulnerability to multiple failure mechanisms, presents significant challenges for SHM throughout their service life. While non-destructive testing techniques have been utilized to address these challenges, they are often hindered by high costs and limited resolution. Furthermore, routine inspections may fail to detect damage occurring between scheduled assessments. Consequently, there is an increasing demand for SHM methods capable of providing continuous monitoring without requiring aircraft grounding.

Taymaz et al. focused on advancing aerospace SHM through the application of piezoresistive MXene fibers [159]. MXene, recognized for its two-dimensional nanostructure and exceptional properties, offers distinct advantages for strain sensing applications. Piezoresistive MXene fibers were successfully fabricated using wet spinning and integrated into carbon fiber-reinforced epoxy laminates to enable in situ strain sensing. The results demonstrate that MXene fibers exhibit remarkable sensitivity within low strain ranges, achieving a maximum gauge factor of 0.9 at 0.13% strain. Additionally, the fibers showed high reliability under repeated tensile deformations and low-velocity impact loading conditions. This research contributes to the development of self-sensing composite materials, enhancing early detection capabilities for damage and defects in aerospace structures, thereby improving safety and reducing maintenance costs [159].

**Figure 12:**
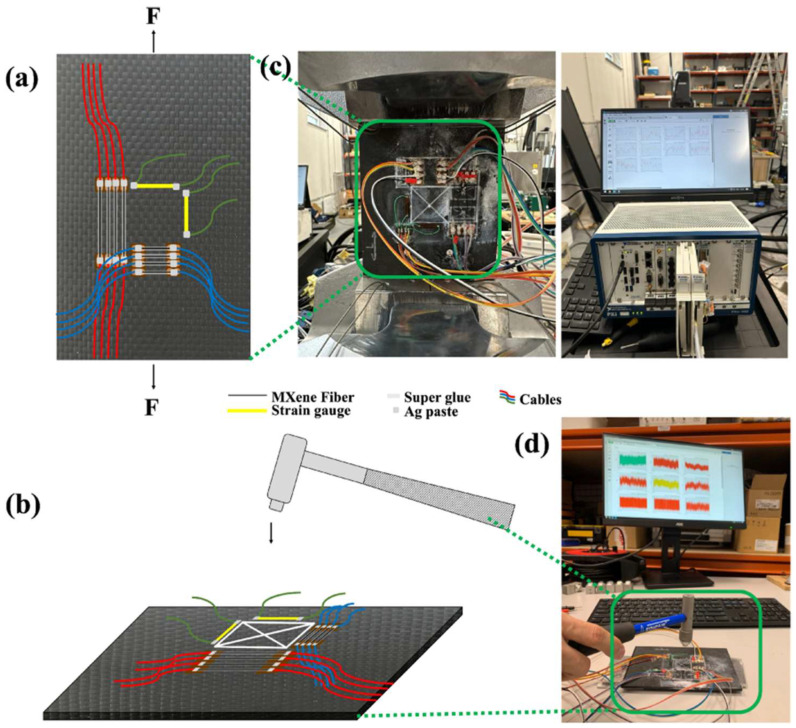
Composite specimens embedded with MXene fibers were prepared for (**a**) cyclic tensile testing and (**b**) low-energy impact testing using a hammer tap. Photographs were captured during both (**c**) the cyclic tensile tests and (**d**) the hammer tap impact tests [159].

A fiber-reinforced composite sample was fabricated by attaching two sets of MXene fibers of different lengths to its surface (Figure 12a,b). Strain sensing performance was evaluated using cyclic tensile and hammer tap impact tests. Cyclic tests were conducted on an MTS Acumen 12 system, with strain measured via attached gauges. A single loading cycle at 60 MPa and 3 Hz, simulating high-G UAV maneuvers, was applied to assess resistance change and gauge factor. Electrical data were recorded using the NI PXI system with PXI-8861, FlexLogger, and PXIe-4339 modules for multichannel resistance monitoring (Figure 12c). For impact testing, at least 50 hammer strikes (100–400 N) were applied at the laminate’s diagonal center (Figure 12). The measurement system was extended with the PXIe-4499 module to capture impact force profiles. Signals from the hammer, MXene fibers, and strain gauges were collected simultaneously at 25 kHz (Figure 12d) [159].

## 6. Challenges and Opportunities

Polymer waveguide sensors hold immense promise due to their inherent advantages, such as low-cost fabrication, mechanical flexibility, and ease of functionalization [36,123,160,161]. However, several key challenges hinder their widespread deployment and long-term viability [162]. A primary concern is long-term stability; polymers tend to degrade over time due to environmental factors such as temperature fluctuations, humidity, UV exposure, and oxidative aging [134,163,164]. The degradation behavior of polymer materials used in photonic sensors varies significantly depending on their chemical structure. For instance, PMMA is prone to photo-oxidative degradation under UV exposure, leading to chain scission and loss of optical clarity [165]. SU-8, a highly crosslinked epoxy, offers good thermal stability but can absorb moisture over time, causing slight dimensional changes or delamination [166]. Polyimides exhibit excellent thermal and chemical resistance but may undergo oxidative degradation at temperatures above 400 °C in the presence of oxygen [71]. PDMS, while flexible and biocompatible, can degrade via siloxane bond cleavage under UV or ozone exposure [167]. Environmental conditions such as high humidity (>80% RH), elevated temperatures (>100 °C), prolonged UV irradiation (>300 nm), or the presence of oxidizing agents accelerate these degradation processes. Therefore, careful material selection and encapsulation strategies are critical for ensuring the long-term stability of polymer-based photonic devices, especially in outdoor or industrial applications.

This environmental sensitivity can lead to drift in optical properties and reduced sensing accuracy, particularly in applications requiring consistent performance over months or years. In parallel, integration with electronics and optical readout systems remains a technical hurdle. Achieving low-loss, scalable coupling between polymer waveguides and standard photonic or optoelectronic components (e.g., photodetectors, modulators) requires precise alignment, compatible materials, and innovative packaging techniques [164,168].

Beyond material degradation and integration issues, packaging remains a critical technical challenge in the deployment of large-area polymer photonic sensors. Due to their inherent flexibility and sensitivity to environmental factors, polymers require packaging solutions that provide mechanical protection, hermetic sealing, and thermal stability without compromising optical performance [169,170]. For example, PDMS-based sensors often need moisture barriers to prevent swelling, while polyimide waveguides may demand thermally conductive encapsulants in high-temperature environments [171]. Achieving robust electrical and optical interfaces between polymer waveguides and conventional electronics also requires precision alignment and stress-relieving structures to prevent delamination or optical losses over time. As such, the development of scalable, low-cost packaging strategies tailored to flexible photonic substrates is a vital step toward reliable real-world deployment [168].

Co-packaged optics increasingly rely on optical interfaces that support high integration density, low loss, and broadband performance while remaining compatible with standard electrical redistribution methods. To meet these demands, Asch et al. demonstrated two heterogeneous integration strategies for establishing efficient optical connections between on-chip silicon nitride (SiN) waveguides and package-level polymer waveguides using adiabatic coupling [162]. In the first method, polymer waveguides were directly patterned onto the photonic chip through conventional lithography, aligning with chip-first fan-out wafer-level packaging workflows. The second method involves flip-chip bonding of the photonic chip to the package substrate, allowing flexible integration for chip-last approaches. Both techniques were experimentally validated, achieving coupling efficiencies of approximately 1 dB in the O-band for both TE and TM polarizations. The SiN waveguides employed tapers designed using the Mono method to enable effective phase matching between the dissimilar waveguides, which is a key factor in achieving low-loss performance. These results demonstrated the viability of polymer waveguides for co-packaged optics, enabling chip-to-chip and chip-to-fiber connections with total losses under 2 dB [169].

Another significant issue is reproducibility in large-scale production. While polymers enable cost-effective fabrication via soft lithography, imprinting, or R2R processes, maintaining uniformity in waveguide dimensions, surface roughness, and refractive index across batches poses a challenge [172,173]. Variations can lead to inconsistencies in performance, which is particularly critical in commercial or biomedical applications. Nevertheless, these challenges also open avenues for innovation. One promising direction is the development of hybrid material systems, combining polymers with functional nanomaterials such as 2D materials (e.g., graphene, MoS_2_) or inorganic platforms like silicon or silicon nitride [174,175]. These hybrid systems can synergistically merge the mechanical and chemical versatility of polymers with the superior optical or electronic properties of inorganic materials, potentially leading to enhanced sensitivity, stability, and functionality [176,177]. The successful integration of such hybrid platforms could bridge the gap between polymer waveguides and established semiconductor technologies, accelerating the development of next-generation optical sensors.

For instance, to realize a compact mode-locked laser, a germanium–polymer hybrid waveguide was positioned between an indium phosphide (InP) reflective gain chip and a fiber Bragg grating (FBG) [175]. This hybrid waveguide enabled efficient optical coupling by matching the mode fields between the gain chip and the fiber. A key component of the structure is a 50-nanometer amorphous germanium (α-Ge) layer, which exhibited nonlinear absorption at 1550 nm. The nonlinear properties of the waveguide were confirmed through femtosecond pulse transmission experiments, which revealed pulse compression effects. By incorporating the hybrid waveguide into the laser cavity, the α-Ge layer functions as a passive saturable absorber, enabling modulation of the cavity’s longitudinal modes to produce pulsed operation. The laser employed the InP chip for its compact gain source and a tunable-length FBG to control the pulse repetition frequency. Experimental results demonstrated stable mode-locking with a repetition rate near 50 MHz, a pulse width of 147 ps, and a signal-to-noise ratio greater than 50 dB. This approach introduced a “ternary” laser architecture combining discrete photonic elements with a polymer-integrated waveguide and establishes α-Ge films as promising low-cost saturable absorbers for integrated photonic systems [175].

## 7. Future Outlook

Polymer photonic devices have demonstrated remarkable potential across a broad spectrum of applications due to their inherent flexibility, low cost, and tunable optical properties. As research and development in this field progress, several emerging directions promise to define the next era of polymer photonic technologies. Among these, four promising avenues stand out: smart packaging with embedded polymer photonic sensors, flexible and wearable large-area photonic sensor arrays, advances in printable photonics and additive manufacturing and polymer photonics for environmental resilience and harsh conditions.

To effectively compare and track the progress of emerging directions in polymer photonic sensing such as smart packaging, wearable sensor arrays, and printable photonics, it is essential to adopt a unified set of evaluation metrics. Key performance indicators may include sensitivity (e.g., detection limit in RIU or analyte concentration), resolution (spatial, spectral, or temporal), fabrication cost per unit area, throughput (e.g., cm^2^/min in R2R processing), mechanical durability (e.g., number of bending cycles to failure), and power consumption for integrated systems. These metrics enable objective comparisons across different materials, device architectures, and application domains. For example, while smart packaging prioritizes low cost and large-area throughput, wearable sensors require high flexibility and biocompatibility, and printable photonics demand design freedom and integration speed. By framing future advances through these quantitative benchmarks, the field can more systematically evaluate trade-offs and drive targeted innovation.

### 7.1. Smart Packaging with Embedded Polymer Photonic Sensors

The convergence of polymer photonics and intelligent packaging technologies offers transformative capabilities in food safety, pharmaceuticals, and logistics [178,179,180]. Smart packaging equipped with polymer-based photonic sensors can provide real-time information about environmental conditions such as temperature, humidity, gas concentration, and even microbial contamination. These sensors typically rely on stimuli-responsive polymers that undergo a reversible change in optical properties such as colorimetric shifts or fluorescence modulation, when exposed to specific analytes [168].

Emerging work on photonic crystals, Bragg stacks, and microring resonators fabricated from responsive polymer matrices shows promise for low-cost integration into packaging films and labels [181,182,183]. In particular, fully passive, battery-free sensors based on structural color shifts or photonic barcodes can allow for seamless, scalable deployment. When combined with QR codes or NFC-enabled data capture, such devices could enable end-to-end tracking and authenticity verification. Future efforts will likely focus on improving the specificity, sensitivity, and shelf stability of these photonic elements, as well as developing sustainable, biodegradable substrates compatible with food-grade regulations [184].

### 7.2. Flexible/Wearable Large-Area Photonic Sensor Arrays

The development of conformal, large-area photonic sensor arrays is poised to revolutionize human health monitoring, soft robotics, and environmental sensing [185]. Polymer materials, by virtue of their mechanical compliance and optical versatility, are well-suited for the creation of wearable photonic devices that can be laminated onto skin or textiles [186,187]. These sensor arrays can monitor parameters such as strain, pressure, hydration, pH, and biomolecular markers with high spatial and temporal resolution [188].

Photonic sensing mechanisms in this context often include waveguide-based interferometers, flexible optical fibers, photonic skins, and surface plasmon-enhanced films [22]. Recent innovations in stretchable and self-healing polymer photonics further extend the durability and reusability of such arrays under dynamic physiological conditions [186]. In tandem with flexible light sources and detectors, fully integrated systems for on-body photonic sensing are within reach [189].

A critical challenge in this domain is the need for scalable fabrication strategies that ensure device uniformity and signal fidelity across large areas. Integration with wireless communication modules and low-power signal processing will also be pivotal for real-world deployment [190]. Moving forward, synergistic development in polymer chemistry, optical engineering, and wearable electronics will play a central role in realizing practical and high-performance photonic wearables [191].

### 7.3. Advances in Printable Photonics and Additive Manufacturing

Additive manufacturing and printing technologies such as inkjet printing, aerosol jet printing, direct laser writing, and NIL are reshaping how polymer photonic devices are conceived and fabricated [192,193,194,195]. These techniques allow for the rapid prototyping and low-cost mass production of complex photonic structures on a wide range of substrates, including flexible and stretchable surfaces [196].

Functional polymer inks, comprising active photonic materials, nanoparticles, or dopants, can be precisely deposited to create multilayered optical circuits, gratings, and resonators [82,98,184,197]. Additive manufacturing also enables the integration of photonic and electronic components within a single fabrication workflow, accelerating the development of optoelectronic systems for sensing, communication, and computing.

One of the most promising frontiers in this area is 3D printing of photonic crystals and gradient refractive index structures using custom-tailored polymer resins [198,199]. These structures can be engineered to exhibit tailored dispersion properties, enabling novel optical functionalities such as slow light, omnidirectional reflectors, and optical cloaking [28]. Moreover, the coupling of additive manufacturing with machine learning-driven design optimization may pave the way for next-generation photonic devices with previously unattainable performance metrics [200,201].

Despite these advances, key challenges remain, including resolution limits, ink stability, and compatibility with multi-material systems. Future research must address these barriers while pushing toward fully integrated, printable polymer photonic platforms suitable for applications ranging from point-of-care diagnostics to intelligent infrastructure.

### 7.4. Polymer Photonics for Environmental Resilience and Harsh Conditions

Future research in polymer photonic sensors will increasingly focus on enhancing material resilience for deployment in extreme or challenging environments. This includes the development of thermally stable, chemically resistant, and UV-hardened polymers capable of maintaining optical performance in high-temperature industrial zones, corrosive chemical plants, or high-radiation outdoor settings [202]. Functional polymers with built-in environmental responsiveness such as self-healing, moisture-resistant, or anti-fouling properties will support long-term reliability and reduced maintenance in field-deployed sensors. Moreover, hybrid polymer-inorganic systems and protective coatings may further extend sensor lifetimes and enable new applications in environmental remediation, early hazard detection, and infrastructure monitoring in harsh climates [203]. These advances will be critical for expanding the reach of low-cost, scalable polymer photonics into domains where durability and environmental compatibility are paramount [204].

## 8. Conclusions

The maturation of polymer photonic technologies marks a significant step toward democratizing access to advanced sensing capabilities. This review has demonstrated that beyond their cost-effectiveness, polymer platforms offer remarkable versatility in material selection, structural design, and functionalization strategies. Their compatibility with unconventional substrates and conformal surfaces supports form factors not achievable with rigid photonic materials, making them uniquely suited for applications in dynamic or space-constrained environments. The reviewed fabrication techniques ranging from nanoimprinting to additive manufacturing showcase the field’s progression toward high-throughput, low-waste production paradigms. Each method presents trade-offs in terms of resolution, scalability, and integration complexity, underscoring the importance of application-specific design choices. Similarly, the diverse range of polymer-based photonic structures reveals how tailored geometries and guided-mode configurations can optimize sensing performance across chemical, biological, mechanical, and environmental domains. Yet, critical bottlenecks persist. These include material aging, environmental sensitivity, and challenges in monolithic integration with electronics and signal processing hardware. Addressing these issues will require multidisciplinary efforts spanning polymer chemistry, photonic design, and packaging technologies. Future progress hinges on the development of robust, reproducible fabrication workflows and the seamless hybridization of polymer photonics with established microelectronic platforms. With sustained innovation, polymer photonic sensors are poised to become foundational components of next-generation intelligent sensing systems.

In the author’s view, the future development of polymer-based photonic sensors can be understood across three distinct time horizons: short term (1–3 years), medium term (3–5 years), and long term (5–10 years), each reflecting current capabilities alongside emerging opportunities. In the short term, the author anticipates that research will primarily focus on enhancing environmental stability, reproducibility, and scalable fabrication techniques. This will be driven by advances in material formulations and manufacturing processes such as roll-to-roll, inkjet, and nanoimprint lithography. Additionally, the author expects significant progress in hybrid polymer-inorganic systems and printable photonics, enabling cost-effective and disposable sensors targeted at healthcare, environmental monitoring, and IoT applications.

Looking toward the medium term, the author foresees the emergence of multifunctional polymer photonic devices that integrate sensing, data processing, and wireless communication. These developments will likely coincide with a stronger convergence between polymer photonics, flexible electronics, and energy harvesting technologies, paving the way for fully autonomous sensor systems. Applications in wearables, smart packaging, and structural health monitoring are expected to expand, fueled by advancements in additive manufacturing and materials science.

In the long term, the author believes the field will evolve towards widespread deployment of biodegradable, sustainable, and fully printed photonic–electronic systems, addressing needs in smart cities, personalized medicine, and environmental sensing. Moreover, it is anticipated that emerging trends such as AI-driven photonic sensing and quantum-enhanced platforms will further broaden the capabilities and impact of polymer photonic technologies.

## Data Availability

No new data are produced.

## References

[1] Butt M.A., Mateos X., Piramidowicz R. (2024). Photonics Sensors: A Perspective on Current Advancements, Emerging Challenges, and Potential Solutions (Invited). Phys. Lett. A.

[2] Butt M.A., Mateos X. (2024). Strategic Insights into Integrated Photonics: Core Concepts, Practical Deployments, and Future Outlook. Appl. Sci..

[3] Zeng F., Pang C., Tang H. (2024). Sensors on Internet of Things Systems for the Sustainable Development of Smart Cities: A Systematic Literature Review. Sensors.

[4] Meriç M.K. (2025). Implementation of a Wireless Sensor Network for Irrigation Management in Drip Irrigation Systems. Sci. Rep..

[5] Javaid M., Haleem A., Singh R.P., Suman R., Gonzalez E.S. (2022). Understanding the Adoption of Industry 4.0 Technologies in Improving Environmental Sustainability. Sustain. Oper. Comput..

[6] Golovastikov N.V., Kazanskiy N.L., Khonina S.N. (2025). Optical Fiber-Based Structural Health Monitoring: Advancements, Applications, and Integration with Artificial Intelligence for Civil and Urban Infrastructure. Photonics.

[7] Fallahi V., Kordrostami Z., Hosseini M. (2024). Sensitivity and Quality Factor Improvement of Photonic Crystal Sensors by Geometrical Optimization of Waveguides and Micro-Ring Resonators Combination. Sci. Rep..

[8] Kazanskiy N.L., Khonina S.N., Butt M.A. (2023). A Review of Photonic Sensors Based on Ring Resonator Structures: Three Widely Used Platforms and Implications of Sensing Applications. Micromachines.

[9] Xu H., Hafezi M., Fan J., Taylor J.M., Strouse G.F., Ahmed Z. (2014). Ultra-Sensitive Chip-Based Photonic Temperature Sensor Using Ring Resonator Structures. Opt. Express.

[10] Butt M.A., Imran Akca B., Mateos X. (2025). Integrated Photonic Biosensors: Enabling Next-Generation Lab-on-a-Chip Platforms. Nanomaterials.

[11] Butt M.A. (2025). Silicon-on-Insulator Coupled Waveguide-Resonator Sensor: Trade-off between Sensitivity and Other Performance Parameters. Opt. Commun..

[12] Badoni D., Gunnella R., Salamon A., Bonaiuto V., Liberali V., Salina G., De Matteis F., Mai A., Salvato M., Colavecchi L. Design and Test of Silicon Photonic Mach-Zehnder Interferometers for Data Transmission Applications. Proceedings of the 2020 Italian Conference on Optics and Photonics (ICOP).

[13] Fehlen P., Thomas G., Posada F.G., Guise J., Rusconi F., Cerutti L., Taliercio T., Spitzer D. (2023). III-V Semiconductor Plasmonics for Gas Sensing of Organophosphorous Compounds. Proceedings of the Quantum Sensing and Nano Electronics and Photonics XIX, San Francisco, CA, USA, 29 January–2 February 2023.

[14] Lijing Z., Zakoldaev R.A., Sergeev M.M., Veiko V.P. (2020). Fluorescent Bulk Waveguide Sensor in Porous Glass: Concept, Fabrication, and Testing. Nanomaterials.

[15] Wen S.M., Chui C.O. (2013). CMOS Junctionless Field-Effect Transistors Manufacturing Cost Evaluation. IEEE Trans. Semicond. Manuf..

[16] Lopez P., Mabe J., Miró G., Etxeberria L. (2018). Low Cost Photonic Sensor for In-Line Oil Quality Monitoring: Methodological Development Process towards Uncertainty Mitigation. Sensors.

[17] Castelló J.G., Toccafondo V., Pérez-Millán P., Losilla N.S., Cruz J.L., Andrés M.V., García-Rupérez J. (2011). Real-Time and Low-Cost Sensing Technique Based on Photonic Bandgap Structures. Opt. Lett..

[18] Dias L., Shoman H., Luan E., Jayatilleka H., Shekhar S., Chrostowski L., Jaeger N.A.F. (2023). Cost-Effective Silicon-Photonic Biosensors Using Doped Silicon Detectors and a Broadband Source. Opt. Express.

[19] Butt M.A., Tyszkiewicz C., Karasiński P., Zięba M., Hlushchenko D., Baraniecki T., Kaźmierczak A., Piramidowicz R., Guzik M., Bachmatiuk A. (2022). Development of a Low-Cost Silica-Titania Optical Platform for Integrated Photonics Applications. Opt. Express.

[20] Khonina S.N., Voronkov G.S., Grakhova E.P., Kazanskiy N.L., Kutluyarov R.V., Butt M.A. (2023). Polymer Waveguide-Based Optical Sensors—Interest in Bio, Gas, Temperature, and Mechanical Sensing Applications. Coatings.

[21] Prasanna Kumaar S., Sivasubramanian A. (2024). Design of a High-Sensitivity Polymer Double-Slot Waveguide Sensor for Point-of-Care Biomedical Applications. Sens. Int..

[22] Nagar M.A., Janner D. (2024). Polymer-Based Optical Guided-Wave Biomedical Sensing: From Principles to Applications. Photonics.

[23] Shao Z., Liu J., Zhou K., Zhang Z., Liang R., Qiao X. (2024). Advanced Fabrication of Polymer Waveguide Interferometric Sensor Utilizing Interconnected Holey Fibers. Opt. Express.

[24] Prajzler V., Chlupaty V., Kulha P., Neruda M., Kopp S., Mühlberger M. (2021). Optical Polymer Waveguides Fabricated by Roll-to-Plate Nanoimprinting Technique. Nanomaterials.

[25] Bruck R., Muellner P., Kataeva N., Koeck A., Trassl S., Rinnerbauer V., Schmidegg K., Hainberger R. (2013). Flexible Thin-Film Polymer Waveguides Fabricated in an Industrial Roll-to-Roll Process. Appl. Opt..

[26] Hiltunen M., Hiltunen J., Stenberg P., Petäjä J., Heinonen E., Vahimaa P., Karioja P. (2012). Polymeric Slot Waveguide at Visible Wavelength. Opt. Lett..

[27] Kocabas A., Aydinli A. (2006). Polymeric Waveguide Bragg Grating Filter Using Soft Lithography. Opt. Express.

[28] Ngo G.L., Nguyen L., Hermier J.-P., Lai N.D. (2023). On-Chip 3D Printing of Polymer Waveguide-Coupled Single-Photon Emitter Based on Colloidal Quantum Dots. Polymers.

[29] Zhang C., Zhao Y.S. (2024). Flexible Photonic Materials and Devices: Synthetic Strategies, Sensing Properties, and Wearable Applications. Adv. Mater..

[30] Paz L.F., Caño-García M., Geday M.A., Otón J.M., Quintana X. (2022). Identification of Dyes and Matrices for Dye Doped Polymer Waveguide Emitters Covering the Visible Spectrum. Sci. Rep..

[31] Li H., Meng W., Cao L., Zhang L., Liu Y., Lin Z., Zhao R., Song Z., Ren F., Zhang S. (2023). Fabrication and Characterization of Polymer Optical Waveguide Bragg Grating for Pulse Signal Sensing. Opt. Express.

[32] Lee E.-S., Chun K.-W., Jin J., Lee S.-S., Oh M.-C. (2023). Monolithic Integration of Polymer Waveguide Phase Modulators with Silicon Nitride Waveguides Using Adiabatic Transition Tapers. Opt. Express.

[33] Park T.-H., Kim S.-M., Oh M.-C. (2019). Polymer-Waveguide Bragg-Grating Devices Fabricated Using Phase-Mask Lithography. Curr. Opt. Photon..

[34] Roth G.-L., Kefer S., Hessler S., Esen C., Hellmann R. (2021). Polymer Photonic Crystal Waveguides Generated by Femtosecond Laser. Laser Photonics Rev..

[35] La T.L., Bui B.N., Nguyen T.T.N., Pham T.L., Tran Q.T., Tong Q.C., Mikulich A., Nguyen T.P., Nguyen T.T.T., Lai N.D. (2023). Design and Realization of Polymeric Waveguide/Microring Structures for Telecommunication Domain. Micromachines.

[36] Jiang L., Wu J., Chen K., Zheng Y., Deng G., Zhang X., Li Z., Chiang K.S. (2020). Polymer Waveguide Mach-Zehnder Interferometer Coated with Dipolar Polycarbonate for on-Chip Nitroaromatics Detection. Sens. Actuators B Chem..

[37] Guo J., Yang C., Dai Q., Kong L. (2019). Soft and Stretchable Polymeric Optical Waveguide-Based Sensors for Wearable and Biomedical Applications. Sensors.

[38] Ahmed I., Ali M., Elsherif M., Butt H. (2023). UV Polymerization Fabrication Method for Polymer Composite Based Optical Fiber Sensors. Sci. Rep..

[39] Desidery L., Lanotte M., Giustozzi F., Nizamuddin S. (2022). 1-Polymers and Plastics: Types, Properties, and Manufacturing. Plastic Waste for Sustainable Asphalt Roads.

[40] Potaufeux J.-E., Odent J., Notta-Cuvier D., Lauro F., Raquez J.-M. (2020). A Comprehensive Review of the Structures and Properties of Ionic Polymeric Materials. Polym. Chem..

[41] Puszka A., Podkościelna B. (2022). Special Issue: Synthesis, Processing, Structure and Properties of Polymer Materials. Polymers.

[42] Sowa S., Watanabe W., Tamaki T., Nishii J., Itoh K. (2006). Symmetric Waveguides in Poly(Methyl Methacrylate) Fabricated by Femtosecond Laser Pulses. Opt. Express.

[43] Zheng L., Keppler N., Zhang H., Behrens P., Roth B. (2022). Planar Polymer Optical Waveguide with Metal-Organic Framework Coating for Carbon Dioxide Sensing. Adv. Mater. Technol..

[44] Nitiss E., Tokmakovs A., Pudzs K., Busenbergs J., Rutkis M. (2017). All-Organic Electro-Optic Waveguide Modulator Comprising SU-8 and Nonlinear Optical Polymer. Opt. Express.

[45] Li H., Wang Y., Sun Y., Zhang S., An Z., Zhang S., Zhang C., Zhang Z., Mao Q., García J.D.P. (2022). Design and Fabrication of SU-8 Polymer Arrayed Waveguide Gratings Based on Flexible PDMS Substrates. Appl. Opt..

[46] Buzzin A., Asquini R., Caputo D., de Cesare G. (2021). On-Glass Integrated SU-8 Waveguide and Amorphous Silicon Photosensor for On-Chip Detection of Biomolecules: Feasibility Study on Hemoglobin Sensing. Sensors.

[47] Xin Y., Pandraud G., Zhang Y., French P. (2019). Single-Mode Tapered Vertical SU-8 Waveguide Fabricated by E-Beam Lithography for Analyte Sensing. Sensors.

[48] Maruno T., Sakata T., Ishii T., Maruo Y.Y., Sasaki S., Tamamura T. (1995). Polyimide Optical Waveguides Fabricated by Direct Electron Beam Writing. Proceedings of the Organic Thin Films for Photonic Applications (1995).

[49] Wessa T., Barié N., Rapp M., Ache H.J. (1998). Polyimide, a New Shielding Layer for Sensor Applications. Sens. Actuators B Chem..

[50] Mavrona E., Graf J., Hack E., Zolliker P. (2021). Optimized 3D Printing of THz Waveguides with Cyclic Olefin Copolymer. Opt. Mater. Express.

[51] Okagbare P.I., Emory J.M., Datta P., Goettert J., Soper S.A. (2010). Fabrication of a Cyclic Olefin Copolymer Planar Waveguide Embedded in a Multi-Channel Poly(Methyl Methacrylate) Fluidic Chip for Evanescence Excitation. Lab. Chip.

[52] Wiesmayr B., Höglinger M., Krieger M., Lindner P., Baumgartner W., Stadler A.T. (2019). A Polydimethylsiloxane (PDMS) Waveguide Sensor That Mimics a Neuromast to Measure Fluid Flow Velocity. Sensors.

[53] Zimmermann C.A., Amouzou K.N., Ung B. (2025). Recent Advances in PDMS Optical Waveguides: Properties, Fabrication, and Applications. Adv. Opt. Mater..

[54] Sarkar S., Poulose S., Sahoo P.K., Joseph J. (2018). Flexible and Stretchable Guided-Mode Resonant Optical Sensor: Single-Step Fabrication on a Surface Engineered Polydimethylsiloxane Substrate. OSA Contin..

[55] Janeiro R., Flores R., Viegas J. (2019). Silicon Photonics Waveguide Array Sensor for Selective Detection of VOCs at Room Temperature. Sci. Rep..

[56] Ahmed K.K., Muheddin D.Q., Mohammed P.A., Ezat G.S., Murad A.R., Ahmed B.Y., Hussen S.A., Ahmed T.Y., Hamad S.M., Abdullah O.G. (2024). A Brief Review on Optical Properties of Polymer Composites: Insights into Light-Matter Interaction from Classical to Quantum Transport Point of View. Results Phys..

[57] Lim J.W. (2024). Polymer Materials for Optoelectronics and Energy Applications. Materials.

[58] Pitois C., Hult A., Wiesmann D. (2001). Absorption and Scattering in Low-Loss Polymer Optical Waveguides. J. Opt. Soc. Am. B.

[59] Rudge A., Davey J., Raistrick I., Gottesfeld S., Ferraris J.P. (1994). Conducting Polymers as Active Materials in Electrochemical Capacitors. J. Power Sources.

[60] Khaleque M.A., Aly Saad Aly M., Khan M.Z.H. (2025). Chemical and Electrochemical Synthesis of Doped Conducting Polymers and Their Application in Supercapacitors: An Overview. Chem. Eng. J..

[61] Khdary N.H., Almuarqab B.T., El Enany G. (2023). Nanoparticle-Embedded Polymers and Their Applications: A Review. Membranes.

[62] Mostafa A.M., Salem A., Al-Ahmadi A.N., Mwafy E.A., Elsharkawy W.B., Nafee S.S., Alshammary A.J., Toghan A., Motawea M.M. (2025). Nickel Oxide Nanoparticles Embedded in Polymer-Matrix Nanocomposite Prepared by Nanosecond Laser Ablation Method for Optoelectronic Applications. Radiat. Phys. Chem..

[63] Rezaei F., Dinari M. (2021). Cu Nanoparticles Embedded in the Porous Organic Polymer as Highly Effective Catalysts for Nitroaromatics Reduction. Microporous Mesoporous Mater..

[64] Imato K., Ooyama Y. (2024). Stimuli-Responsive Smart Polymers Based on Functional Dyes. Polym. J..

[65] Milvich J., Kohler D., Freude W., Koos C. (2018). Surface Sensing with Integrated Optical Waveguides: A Design Guideline. Opt. Express.

[66] Bhatia Y., Zheng L., Steinbach L., Günther A., Schneider A., Roth B. (2025). Low-Cost Scalable Fabrication of Functionalized Optical Waveguide Arrays for Gas Sensing Application. Int. J. Adv. Manuf. Technol..

[67] Sharma K., Mohammed W.S., Bora T. (2025). Development of Methacrylate-Based Polymer Waveguides as an Optical Sensing Element. Proceedings of the Optical Waveguide and Laser Sensors IV.

[68] Myndrul V., Arreguin-Campos R., Iatsunskyi I., Di Scala F., Eersels K., van Grinsven B. (2025). Photonic Sensor Based on Surface Imprinted Polymers for Enhanced Point-of-Care Diagnosis of Bacterial Urinary Tract Infections. Biosens. Bioelectron..

[69] Ting Y.-C., Shy S.-L. (2016). Nano-Imprint Lithography Using Poly (Methyl Methacrylate) (PMMA) and Polystyrene (PS) Polymers. Proceedings of the Alternative Lithographic Technologies VIII.

[70] Eryürek M., Tasdemir Z., Karadag Y., Anand S., Kilinc N., Alaca B.E., Kiraz A. (2017). Integrated Humidity Sensor Based on SU-8 Polymer Microdisk Microresonator. Sens. Actuators B Chem..

[71] Zhang T., Chai Y., Wang S., Yu J., Jiang S., Zhu W., Fang Z., Li B. (2023). Recent Study Advances in Flexible Sensors Based on Polyimides. Sensors.

[72] Liedert C., Rannaste L., Kokkonen A., Huttunen O.-H., Liedert R., Hiltunen J., Hakalahti L. (2020). Roll-to-Roll Manufacturing of Integrated Immunodetection Sensors. ACS Sens..

[73] Chen J., Zheng J., Gao Q., Zhang J., Zhang J., Omisore O.M., Wang L., Li H. (2018). Polydimethylsiloxane (PDMS)-Based Flexible Resistive Strain Sensors for Wearable Applications. Appl. Sci..

[74] Lee J.K., Lee T.S. (2005). Newly Synthesized Polybenzoxazole Derivative with an Adjacent Hydroxyphenyl Ring for Optical Sensing. J. Polym. Sci. Part. A Polym. Chem..

[75] Zaitsev N.K., Melnikov P.V., Alferov V.A., Kopytin A.V., German K.E. (2016). Stable Optical Oxygen Sensing Material Based on Perfluorinated Polymer and Fluorinated Platinum(II) and Palladium(II) Porphyrins. Procedia Eng..

[76] ORMOCER® at Fraunhofer ISC. https://www.ormocere.de/en.html.

[77] Hu F., Lin N., Liu X.Y. (2020). Interplay between Light and Functionalized Silk Fibroin and Applications. iScience.

[78] Anantha-Iyengar G., Shanmugasundaram K., Nallal M., Lee K.-P., Whitcombe M.J., Lakshmi D., Sai-Anand G. (2019). Functionalized Conjugated Polymers for Sensing and Molecular Imprinting Applications. Prog. Polym. Sci..

[79] Butt M.A. (2023). Integrated Optics: Platforms and Fabrication Methods. Encyclopedia.

[80] Park J., Lee K.-T., Yeon G., Choi J., Kim M., Han B., Baac H.W., Guo L.J., Ok J.G. (2021). Demonstration of the One-Step Continuous Fabrication of Flexible Polymer Ridge Waveguides via Nanochannel-Guided Lithography. J. Ind. Eng. Chem..

[81] Diez M., Raimbault V., Joly S., Oyhenart L., Doucet J.B., Obieta I., Dejous C., Bechou L. (2018). Direct Patterning of Polymer Optical Periodic Nanostructures on CYTOP for Visible Light Waveguiding. Opt. Mater..

[82] Han X.-Y., Wu Z.-L., Yang S.-C., Shen F.-F., Liang Y.-X., Wang L.-H., Wang J.-Y., Ren J., Jia L.-Y., Zhang H. (2018). Recent Progress of Imprinted Polymer Photonic Waveguide Devices and Applications. Polymers.

[83] Kim W., Yoon G., Kim J., Jeong H., Kim Y., Choi H., Badloe T., Rho J., Lee H. (2022). Thermally-Curable Nanocomposite Printing for the Scalable Manufacturing of Dielectric Metasurfaces. Microsyst. Nanoeng..

[84] Yu G., Mao X., Ding H., Yang F., Wang X. (2024). Inverse-Designed Polarization-Insensitive Metasurface Holography Fabricated by Nanoimprint Lithography. Opt. Lett..

[85] Choi C.-G., Han Y.-T., Kim J.T. Application of UV Nanoimprint Lithography in Polymer Photonic Nano-Systems. Proceedings of the 2006 IEEE Nanotechnology Materials and Devices Conference.

[86] (2020). OrmoCore and OrmoClad—Microresist.

[87] Khan M.U., Justice J., Petäjä J., Korhonen T., Boersma A., Wiegersma S., Karppinen M., Corbett B. (2015). Multi-Level Single Mode 2D Polymer Waveguide Optical Interconnects Using Nano-Imprint Lithography. Opt. Express.

[88] Chuang W.-C., Ho C.-T., Chang W.-C. (2006). Fabrication of Polymer Waveguides by a Replication Method. Appl. Opt..

[89] Huang Y., Paloczi G.T., Scheuer J., Yariv A. (2003). Soft Lithography Replication of Polymeric Microring Optical Resonators. Opt. Express.

[90] Rolland J.P., Hagberg E.C., Denison G.M., Carter K.R., De Simone J.M. (2004). High-Resolution Soft Lithography: Enabling Materials for Nanotechnologies. Angew. Chem. Int. Ed..

[91] Moran I.W., Cheng D.F., Jhaveri S.B., Carter K.R. (2007). High-Resolution Soft Lithography of Thin Film Resists Enabling Nanoscopic Pattern Transfer. Soft Matter.

[92] Poon J.K.S., Huang Y., Paloczi G.T., Yariv A. (2004). Soft Lithography Replica Molding of Critically Coupled Polymer Microring Resonators. IEEE Photonics Technol. Lett..

[93] Bruck R., Muellner P., Kataeva N., Koeck A., Hainberger R., Trassl S., Rinnerbauer V., Schmidegg K. Roll-to-Roll Fabrication of Thin Foil-Based Optical Waveguides with Grating Couplers. Proceedings of the 2012 17th Opto-Electronics and Communications Conference.

[94] Yu S., Zuo H., Gu T., Hu J. A Flexible Polymer Waveguide Platform with Low-Loss Optical Interfaces. Proceedings of the 2021 Conference on Lasers and Electro-Optics Europe & European Quantum Electronics Conference (CLEO/Europe-EQEC).

[95] Kronenfeld J.M., Rother L., Saccone M.A., Dulay M.T., DeSimone J.M. (2024). Roll-to-Roll, High-Resolution 3D Printing of Shape-Specific Particles. Nature.

[96] Lee J., Kim J.Y., Choi J.H., Ok J.G., Kwak M.K. (2017). Scalable Fabrication of Flexible Microstencils by Using Sequentially Induced Dewetting Phenomenon. ACS Omega.

[97] Wolfer T., Bollgruen P., Mager D., Overmeyer L., Korvink J.G. (2014). Flexographic and Inkjet Printing of Polymer Optical Waveguides for Fully Integrated Sensor Systems. Procedia Technol..

[98] Lin C., Jia X., Chen C., Yang C., Li X., Shao M., Yu Y., Zhang Z. (2023). Direct Ink Writing 3D-Printed Optical Waveguides for Multi-Layer Interconnect. Opt. Express.

[99] Butt M.A. (2022). Thin-Film Coating Methods: A Successful Marriage of High-Quality and Cost-Effectiveness—A Brief Exploration. Coatings.

[100] Sreenivasan S.V. (2017). Nanoimprint Lithography Steppers for Volume Fabrication of Leading-Edge Semiconductor Integrated Circuits. Microsyst. Nanoeng..

[101] Moon C.H., Han K.-S., Kim M., Oh D.K., Yi S., Kim T., Kim H., Hwang J., Nam J.G., Lee D.-E. (2023). Scaling up the Sub-50 Nm-Resolution Roll-to-Roll Nanoimprint Lithography Process via Large-Area Tiling of Flexible Molds and Uniform Linear UV Curing. J. Mech. Sci. Technol..

[102] Klestova A., Cheplagin N., Keller K., Slabov V., Zaretskaya G., Vinogradov A.V. (2019). Inkjet Printing of Optical Waveguides for Single-Mode Operation. Adv. Opt. Mater..

[103] Theiler P.M., Lütolf F., Ferrini R. (2018). Non-Contact Printing of Optical Waveguides Using Capillary Bridges. Opt. Express.

[104] Guo L.J. (2007). Nanoimprint Lithography: Methods and Material Requirements. Adv. Mater..

[105] Xia Y., Whitesides G.M. (1998). Soft Lithography. Angew. Chem. Int. Ed..

[106] Yi P., Wu H., Zhang C., Peng L., Lai X. (2015). Roll-to-Roll UV Imprinting Lithography for Micro/Nanostructures. J. Vac. Sci. Technol. B.

[107] Kamyshny A., Magdassi S. (2014). Conductive Nanomaterials for Printed Electronics. Small.

[108] Ngo T.D., Kashani A., Imbalzano G., Nguyen K.T.Q., Hui D. (2018). Additive Manufacturing (3D Printing): A Review of Materials, Methods, Applications and Challenges. Compos. Part. B Eng..

[109] Malheiros-Silveira G.N., Finardi C.A., Van Etten E.A.M.A., Bürger T.S., da Silva R.C.G., Daltrini A.M., Panepucci R.R. Foundry Polymer-Based Inverted-Rib Waveguides. Proceedings of the 2018 SBFoton International Optics and Photonics Conference (SBFoton IOPC).

[110] Prajzler V., Nekvindova P., Hyps P., Brychta J., Jerabek V. (2015). Polymer Planar Optical Waveguides for Optical Interconnections. Proceedings of the 2015 Conference on Lasers and Electro-Optics Pacific Rim (2015).

[111] Butt M.A. (2025). Emerging Trends in Thermo-Optic and Electro-Optic Materials for Tunable Photonic Devices. Materials.

[112] Prasanna Kumaar S., Sivasubramanian A. (2023). Analysis of BCB and SU 8 Photonic Waveguide in MZI Architecture for Point-of-Care Devices. Sens. Int..

[113] Han J., Wu X., Ge X., Xie Y., Song G., Liu L., Yi Y. (2022). Highly Sensitive Liquid M-Z Waveguide Sensor Based on Polymer Suspended Slot Waveguide Structure. Polymers.

[114] Bettotti P., Pitanti A., Rigo E., De Leonardis F., Passaro V.M.N., Pavesi L. (2011). Modeling of Slot Waveguide Sensors Based on Polymeric Materials. Sensors.

[115] Hiltunen M., Hiltunen J., Stenberg P., Aikio S., Kurki L., Vahimaa P., Karioja P. (2014). Polymeric Slot Waveguide Interferometer for Sensor Applications. Opt. Express.

[116] Broadway C., Min R., Leal-Junior A.G., Marques C., Caucheteur C. (2019). Toward Commercial Polymer Fiber Bragg Grating Sensors: Review and Applications. J. Light. Technol..

[117] Ngiejungbwen L.A., Hamdaoui H., Chen M.-Y. (2024). Polymer Optical Fiber and Fiber Bragg Grating Sensors for Biomedical Engineering Applications: A Comprehensive Review. Opt. Laser Technol..

[118] Goraus M., Pudis D., Urbancova P., Martincek I., Gaso P. (2018). Surface-Relief Bragg Grating Waveguides Based on IP-Dip Polymer for Photonic Applications. Appl. Surf. Sci..

[119] Smirnova O., Sajzew R., Finkelmeyer S.J., Asadov T., Chattopadhyay S., Wieduwilt T., Reupert A., Presselt M., Knebel A., Wondraczek L. (2024). Micro-Optical Elements from Optical-Quality ZIF-62 Hybrid Glasses by Hot Imprinting. Nat. Commun..

[120] Nambiar S., P. V., Singh R., Rawat P., Selvaraja S.K. (2024). High-Efficiency Broadband out-of-Plane Fiber-to-Polymer Waveguide Grating Coupler. Opt. Lett..

[121] Wang W., Wu J., Chen K., Jin W., Chiang K.S. (2017). Ultra-Broadband Mode Conversion with Length-Apodized Long-Period Grating on Polymer Waveguide. Proceedings of the Frontiers in Optics 2017 (2017).

[122] Lin H., Xing Y., Chen X., Zhang S., Forsberg E., He S. (2022). Polymer-Based Planar Waveguide Chirped Bragg Grating for High-Resolution Tactile Sensing. Opt. Express.

[123] Zhang Z., Abdalwareth A., Flachenecker G., Angelmahr M., Schade W. (2024). Polymer Waveguide Sensor Based on Evanescent Bragg Grating for Lab-on-a-Chip Applications. Sensors.

[124] Ameen A.A., Panda A., Mehaney A., Almawgani A.H.M., Pradhan D.D., Ali G.A., Ali Y.A.A., Elsayed H.A. (2023). An Investigation of High-Performance Pressure Sensor Employing a Polymer-Defect-Based 1D Annular Photonic Crystal. Photonics.

[125] Hermannsson P.G., Sørensen K.T., Vannahme C., Smith C.L.C., Klein J.J., Russew M.-M., Grützner G., Kristensen A. (2015). All-Polymer Photonic Crystal Slab Sensor. Opt. Express.

[126] Sun J., Maeno K., Aki S., Sueyoshi K., Hisamoto H., Endo T. (2018). Design and Fabrication of a Visible-Light-Compatible, Polymer-Based Photonic Crystal Resonator and Waveguide for Sensing Applications. Micromachines.

[127] Golvari P., Kuebler S.M. (2021). Fabrication of Functional Microdevices in SU-8 by Multi-Photon Lithography. Micromachines.

[128] Mancuso M., Goddard J.M., Erickson D. (2012). Nanoporous Polymer Ring Resonators for Biosensing. Opt. Express.

[129] Madani A., Azarinia H., Latifi H. (2013). Design and Fabrication of a Polymer Micro Ring Resonator with Novel Optical Material at Add/Drop Geometry Using Laser Beam Direct Write Lithography Technique. Optik.

[130] Girault P., Lorrain N., Poffo L., Guendouz M., Lemaitre J., Carré C., Gadonna M., Bosc D., Vignaud G. (2015). Integrated Polymer Micro-Ring Resonators for Optical Sensing Applications. J. Appl. Phys..

[131] Zhang C., Ling T., Chen S.-L., Guo L.J. (2014). Ultrabroad Bandwidth and Highly Sensitive Optical Ultrasonic Detector for Photoacoustic Imaging. ACS Photonics.

[132] Tu X., Chen S.-L., Song C., Huang T., Guo L.J. (2019). Ultrahigh Q Polymer Microring Resonators for Biosensing Applications. IEEE Photonics J..

[133] Xiao Y., Hofmann M., Wang Z., Sherman S., Zappe H. (2016). Design of All-Polymer Asymmetric Mach–Zehnder Interferometer Sensors. Appl. Opt..

[134] Liu F., Zhang X., Wang T., Huang G. (2025). Development and Characterization of an Asymmetric MZI Temperature Sensor Using Polymer Waveguides for Extended Temperature Measurement Scopes. Photonics.

[135] Ma X.X., Chen K.X., Wu J.Y. (2021). Cost-Effective Mach-Zehnder Interferometer Liquid Refractive Index Sensor Based on Conventional Polymer Strip Waveguide. IEEE Photonics J..

[136] Chen M.-Q., Lin Z.-Y., Zhao Y. (2023). Femtosecond Laser Direct-Writing On-Chip MZI Temperature Sensor Based on Polymer Waveguides. IEEE Trans. Instrum. Meas..

[137] Hofmann M., Xiao Y., Sherman S., Gleissner U., Schmidt T., Zappe H. (2016). Asymmetric Mach–Zehnder Interferometers without an Interaction Window in Polymer Foils for Refractive Index Sensing. Appl. Opt..

[138] Du B., Mu X., Liu S., Guo L., Liu Z., Feng S., Xu J., Tong Z., Qi Z.-M. (2022). A New Strategy for Real-Time Humidity Detection: Polymer-Coated Optical Waveguide Sensor. Chemosensors.

[139] Wang J., Yang X., Kou Y., Tong D., Wang A., Niu C., Meng H., Li S., Geng T., Sun W. (2023). Highly-Sensitive Temperature Sensor Based on Photopolymerized-Waveguide Embedded Mach-Zehnder Interferometer. Opt. Express.

[140] Bamiedakis N., Hutter T., Penty R.V., White I.H., Elliott S.R. (2013). PCB-Integrated Optical Waveguide Sensors: An Ammonia Gas Sensor. J. Light. Technol..

[141] Lee S., Lee E.-H., Lee S.-W. (2022). A Flexible and Attachable Colorimetric Film Sensor for the Detection of Gaseous Ammonia. Biosensors.

[142] Yeo J.-E., Ko J.H., Lee S.H., Song Y.M. (2025). Wearable Image-Based Colorimetric Sensor for Real-Time Gas Detection with High Chromaticity. Adv. Electron. Mater..

[143] Chou P.-C., Chen S.-H., Chang C.-J., Lu C.-H., Chen J.-K. (2022). Detection of Heavy Metal Ion Using Photonic Crystals of Polymer Brushes with Reflective Laser Beam System. Appl. Surf. Sci..

[144] Fenzl C., Kirchinger M., Hirsch T., Wolfbeis O.S. (2014). Photonic Crystal-Based Sensing and Imaging of Potassium Ions. Chemosensors.

[145] Das A., Babu A., Chakraborty S., Van Guyse J.F.R., Hoogenboom R., Maji S. (2024). Poly(N-Isopropylacrylamide) and Its Copolymers: A Review on Recent Advances in the Areas of Sensing and Biosensing. Adv. Funct. Mater..

[146] Irawan R., Cheng Y.H., Ng W.M., Aung M.M., Lao I.K., Thaveeprungsriporn V. (2011). Polymer Waveguide Sensor for Early Diagnostic and Wellness Monitoring. Biosens. Bioelectron..

[147] Yi P.F., Shen P., Zheng Y., Chen C.M., Liang L.G., Wang J.H., Guan L.C.S., Zhang D.M. (2017). Metal-Printing Directly Defined Polymer Optical Waveguide Sensors for Detecting Effective Anti-Inflammatory Concentrations of Peimine as Fritillaria Alkaloid Drugs. Opt. Mater. Express.

[148] Cicala G., Arcadio F., Zeni L., Saitta L., Tosto C., Fragalà M.E., Del Prete D., Cennamo N. Plasmonic Sensors Based on 3D-Printed Polymer Waveguides Covered by a Metals Bilayer. Proceedings of the 2022 IEEE Sensors Applications Symposium (SAS).

[149] Zhou Y., Xu Y., Xu G., Sugihara O., Cai B. (2022). Molecularly Imprinted Polymer-Coated Optical Waveguide for Attogram Sensing. ACS Appl. Mater. Interfaces.

[150] Walter J.-G., Alwis L.S.M., Roth B., Bremer K. (2020). All-Optical Planar Polymer Waveguide-Based Biosensor Chip Designed for Smartphone-Assisted Detection of Vitamin D. Sensors.

[151] Pozo F., Tibaduiza D.A., Vidal Y. (2021). Sensors for Structural Health Monitoring and Condition Monitoring. Sensors.

[152] Tanusha D., Badhulika S. (2024). Comparative Analysis of Micro Patterned PDMS-Based Piezoresistive Pressure Sensors with Multifunctional Strain and Health Monitoring Applications. Sens. Actuators A Phys..

[153] Velázquez-Carreón F., Pérez-Alonzo A., Sandoval-Romero G.E., Sánchez-Pérez C. (2024). Enhanced PDMS-Embedded FBG Devices for Displacement Sensing. Opt. Laser Technol..

[154] Zubia J., García I., Villatoro J., Illarramendi M.A., Mateo J., Vázquez C. Polymer Optical Fiber Sensors for Aircraft Structural and Engine Health Monitoring. Proceedings of the 2017 19th International Conference on Transparent Optical Networks (ICTON).

[155] López-Higuera J.M., Cobo L.R., Incera A.Q., Cobo A. (2011). Fiber Optic Sensors in Structural Health Monitoring. J. Light. Technol..

[156] Soman R., Wee J., Peters K. (2021). Optical Fiber Sensors for Ultrasonic Structural Health Monitoring: A Review. Sensors.

[157] Mizuno Y., Theodosiou A., Kalli K., Liehr S., Lee H., Nakamura K. (2021). Distributed Polymer Optical Fiber Sensors: A Review and Outlook. Photon. Res..

[158] Liehr S., Mukhopadhyay S.C. (2011). Polymer Optical Fiber Sensors in Structural Health Monitoring. New Developments in Sensing Technology for Structural Health Monitoring.

[159] Taymaz B.H., Kamış H., Dziendzikowski M., Kowalczyk K., Dragan K., Eskizeybek V. (2025). Enhancing Structural Health Monitoring of Fiber-Reinforced Polymer Composites Using Piezoresistive Ti_3_C_2_T_x_ MXene Fibers. Sci. Rep..

[160] Upadhyay A., Yadav C.S., Maurya R., Sharma G., Singh T.S., Kumar S., Singh V. (2023). Experimental Detection of Chlorpyrifos by MoS_2_ Coated Planar Polymer Waveguide Sensor Utilizing Common Path Interferometric Principle. Optik.

[161] Chen H., Quan W., Zou P., Fu H., Lin Z., Liu X., Xue J., Fan W., Zhang D. Polymer Waveguide Sensor Based on Localized Surface Plasmon Resonance for NaCl Solution Detection. Proceedings of the 2019 IEEE 13th International Conference on Anti-counterfeiting, Security, and Identification (ASID).

[162] Liu F., Guidotti D., Sundaram V., Mahajan S., Huang Z., Chang Y.-J., Chang G.K., Tummala R.R. Material and Process Challenges in Embedding Polymeric Waveguides and Detectors in System on Package (SOP). Proceedings of the 9th International Symposium on Advanced Packaging Materials: Processes, Properties and Interfaces. 2004 Proceedings.

[163] Grandes J., Illarramendi M.A., Arrospide E., Bikandi I., Aramburu I., Guarrotxena N., García O., Zubia J. (2022). Temperature Effects on the Emission of Polymer Optical Fibers Doped with Lumogen Dyes. Opt. Fiber Technol..

[164] Lindsay G.A., Guenthner A.J., Wright M.E., Sanghadasa M., Ashley P.R. (2007). Multi-Month Thermal Aging of Electro-Optic Polymer Waveguides: Synthesis, Fabrication, and Relaxation Modeling. Polymer.

[165] Fukuda Y., Osawa Z. (1991). Wavelength Effect on the Photo-Degradation of Polycarbonate and Poly(Methyl Methacrylate)—Confirmation of the Photo-Degradation Mechanism of PC/PMMA Blends. Polym. Degrad. Stab..

[166] Robin C.J., Jonnalagadda K.N. (2016). Effect of Size and Moisture on the Mechanical Behavior of SU-8 Thin Films. J. Micromech. Microeng..

[167] Song H., Rodriguez N.A., Oakdale J.S., Duoss E.B., Crawford R.H., Seepersad C.C. (2023). Aging of UV Curable PDMS Developed for Large-Scale, High Viscosity Stereolithography. Polym. Degrad. Stab..

[168] Imamura S. Polymeric Optical Waveguides [Materials, Packaging and Applications]. Proceedings of the 1998 IEEE/LEOS Summer Topical Meeting. Digest. Broadband Optical Networks and Technologies: An Emerging Reality. Optical MEMS. Smart Pixels. Organic Optics and Optoelectronics (Cat. No.98TH8369).

[169] Asch J.V., Missinne J., He J., Podpod A., Lepage G., Golshani N., Magdziak R., Sar H., Kobbi H., Bipul S. (2025). Low-Loss Integration of High-Density Polymer Waveguides with Silicon Photonics for Co-Packaged Optics. Optica.

[170] Datta-Chaudhuri T., Abshire P., Smela E. (2014). Packaging Commercial CMOS Chips for Lab on a Chip Integration. Lab. Chip.

[171] Kim M., Huang Y., Choi K., Hidrovo C.H. (2014). The Improved Resistance of PDMS to Pressure-Induced Deformation and Chemical Solvent Swelling for Microfluidic Devices. Microelectron. Eng..

[172] Suda S., Noriki A., Kuwatsuka H., Nakamura F., Atsumi Y., Kurosu T., Murao T., Amano T. (2025). High-Power Stability and Reliability of Polymer Optical Waveguide for Co-Packaged Optics. J. Light. Technol..

[173] Perevoznik D., Tajalli A., Zuber D., Pätzold W., Demircan A., Morgner U. (2021). Writing 3D Waveguides with Femtosecond Pulses in Polymers. J. Light. Technol..

[174] Liu J., Ding Z., Zhang Z. (2024). Ge-Polymer Bridge Waveguide for Mode-Locked Laser Pulse Generation. Opt. Lett..

[175] Zhang Z., Felipe D., Katopodis V., Groumas P., Kouloumentas C., Avramopoulos H., Dupuy J.-Y., Konczykowska A., Dede A., Beretta A. (2015). Hybrid Photonic Integration on a Polymer Platform. Photonics.

[176] ShangGuan L., Zhang D., Zhang T., Cheng R., Wang J., Wang C., Wang F., Ho S.-T., Chen C., Fei T. (2020). Functionalized Polymer Waveguide Optical Switching Devices Integrated with Visible Optical Amplifiers Based on an Organic Gain Material. Dye. Pigment..

[177] Liu J., Zhang Z. (2023). Polymer-Embedding Germanium Nanostrip Waveguide of High Polarization Extinction. Polymers.

[178] Escher A., Megahd H., Tavella C., Comoretto D., Lova P. (2023). Colorimetric Polymer Sensors for Smart Packaging. Macromol. Chem. Phys..

[179] Rodrigues C., Souza V.G.L., Coelhoso I., Fernando A.L. (2021). Bio-Based Sensors for Smart Food Packaging—Current Applications and Future Trends. Sensors.

[180] Ibrahim S., Fahmy H., Elkhawas K., Labeeb A. (2025). Smart Packaging Materials Based Nanoencapsulated Bromothymol as Monitoring Sensors for Spoilage of Chilled Fillet. J. Food Sci. Technol..

[181] Hoffmann G.-A., Wienke A., Reitberger T., Franke J., Kaierle S., Overmeyer L. (2020). Thermoforming of Planar Polymer Optical Waveguides for Integrated Optics in Smart Packaging Materials. J. Mater. Process. Technol..

[182] Lytel R., Lipscomb G.F. (1996). Packaging and Applications of Active Polymer Optical Switching Arrays. Optoelectronic Interconnects and Packaging: A Critical Review.

[183] Flöry N., Halter M., Strässle V., Betschon F., Alexoudi T., Charalampos Z., Lamprecht T. Highly Reliable Polymer Waveguide Platform for Multi-Port Photonic Chip-Packaging. Proceedings of the 2021 IEEE 71st Electronic Components and Technology Conference (ECTC).

[184] Evertz A., Pleuß J., Reitz B., Overmeyer L. (2024). Flexo-Printed Polymer Waveguides for Integration in Electro-Optical Circuit Boards. Flex. Print. Electron..

[185] Kazanskiy N.L., Khonina S.N., Butt M.A. (2024). A Review on Flexible Wearables—Recent Developments in Non-Invasive Continuous Health Monitoring. Sens. Actuators A Phys..

[186] Zha B., Wang Z., Ma L., Chen J., Wang H., Li X., Kumar S., Min R. (2024). Intelligent Wearable Photonic Sensing System for Remote Healthcare Monitoring Using Stretchable Elastomer Optical Fiber. IEEE Internet Things J..

[187] Li H., Li X., Yang Y., Xie F., Han M., Lin Z., Wang Y., Zhang J., Zhang S., Zhang C. (2025). Photonic Skin for Photonic-Integration-Based Wearable Sensors. Optica.

[188] Song R., Cho S., Khan S., Park I., Gao W. (2025). Lighting the Path to Precision Healthcare: Advances and Applications of Wearable Photonic Sensors. Adv. Mater..

[189] Guo J., Tuo J., Sun J., Li Z., Guo X., Chen Y., Cai R., Zhong J., Xu L. (2025). Stretchable Multimodal Photonic Sensor for Wearable Multiparameter Health Monitoring. Adv. Mater..

[190] Khonina S.N., Kazanskiy N.L. (2025). Trends and Advances in Wearable Plasmonic Sensors Utilizing Surface-Enhanced Raman Spectroscopy (SERS): A Comprehensive Review. Sensors.

[191] Ajeev A., Warfle T., Maslaczynska-Salome S., Alipoori S., Duprey C., Wujcik E.K. (2025). From the Synthesis of Wearable Polymer Sensors to Their Potential for Reuse and Ultimate Fate. Chem. Sci..

[192] Hamjah M.-K., Zeitler J., Eiche Y., Lorenz L., Backhaus C., Hoffmann G.-A., Wienke A., Kaierle S., Overmeyer L., Lindlein N. Manufacturing of Polymer Optical Waveguides for 3D-Opto-MID: Review of the OPTAVER Process. Proceedings of the 2021 14th International Congress Molded Interconnect Devices (MID).

[193] Backhaus C., Hoffmann G.A., Reitberger T., Eiche Y., Overmeyer L., Franke J., Lindlein N. (2020). Analysis of Additive Manufactured Polymer Optical Waveguides: Measurement and Simulation of Their Waviness. Proceedings of the Integrated Optics: Devices, Materials, and Technologies XXIV.

[194] Ding Z., Wang H., Li T., Ouyang X., Shi Y., Zhang A.P. (2022). Fabrication of Polymer Optical Waveguides by Digital Ultraviolet Lithography. J. Light. Technol..

[195] Jradi S., Soppera O., Lougnot D.J. (2008). Fabrication of Polymer Waveguides between Two Optical Fibers Using Spatially Controlled Light-Induced Polymerization. Appl. Opt..

[196] Ma Q., Dong K., Li F., Jia Q., Tian J., Yu M., Xiong Y. (2025). Additive Manufacturing of Polymer Composite Millimeter-Wave Components: Recent Progress, Novel Applications, and Challenges. Polym. Compos..

[197] Prajzler V., Chlupaty V., Neruda M. (2022). Circular Large Core Optical Elastomer Waveguides Fabricated by Using Direct Microdispense Fabrication Method. Optik.

[198] Baghdasaryan T., Vanmol K., Berghmans F., Thienpont H., Van Erps J. 3D Printing of Fiber and Waveguide Coupling Components in Polymer. Proceedings of the 2023 International Workshop on Fiber Optics on Access Networks (FOAN).

[199] Trunin P., Cafiso D., Beccai L. (2025). Design and 3D Printing of Soft Optical Waveguides towards Monolithic Perceptive Systems. Addit. Manuf..

[200] Hamjah M.-K., Thielen N., Hagelloch J.E., Franke J. Machine Learning Approach towards Quality Control of Aerosol-Jet Printed Polymer Optical Waveguides Material. Proceedings of the 2021 IEEE Region 10 Symposium (TENSYMP).

[201] Najeeb J., Shah S.S.A., Tahir M.H., Hanafy A.I., El-Bahy S.M., El-Bahy Z.M. (2024). Machine Learning Assisted Designing of Polymers and Refractive Index Prediction: Easy and Fast Screening of Polymers from Chemical Space. Mater. Chem. Phys..

[202] Raju B., Kumar R., Dhanalakshmi S., Hrbac R., Demel L., R. N. (2025). Experimental Investigation on Polymer Coated Fibre Bragg Grating Sensor for Temperature Measurement in Sewer Environment. Results Eng..

[203] Abtahi S., Hendeniya N., Mahmud S.T., Mogbojuri G., Iheme C.L., Chang B. (2025). Metal-Coordinated Polymer–Inorganic Hybrids: Synthesis, Properties, and Application. Polymers.

[204] Butt M.A., Piramidowicz R. (2024). Integrated Photonic Sensors for the Detection of Toxic Gasses—A Review. Chemosensors.

